# Identification of histone deacetylase genes in *Dendrobium officinale* and their expression profiles under phytohormone and abiotic stress treatments

**DOI:** 10.7717/peerj.10482

**Published:** 2020-12-15

**Authors:** Mingze Zhang, Jaime A. Teixeira da Silva, Zhenming Yu, Haobin Wang, Can Si, Conghui Zhao, Chunmei He, Jun Duan

**Affiliations:** 1 Key Laboratory of South China Agricultural Plant Molecular Analysis and Gene Improvement, South China Botanical Garden, Chinese Academy of Sciences, Guangzhou, China; 2 College of Life Sciences, University of Chinese Academy of Sciences, Beijing, China; 3 Independent Researcher, Miki, Kagawa, Japan

**Keywords:** *Dendrobium officinale*, Histone deacetylation, Phylogenetic analysis, Subcellular localization, Stress

## Abstract

The deacetylation of core histones controlled by the action of histone deacetylases (HDACs) plays an important role in the epigenetic regulation of plant gene transcription. However, no systematic analysis of *HDAC* genes in *Dendrobium officinale*, a medicinal orchid, has been performed. In the current study, a total of 14 histone deacetylases in *D. officinale* were identified and characterized using bioinformatics-based methods. These genes were classified into RPD3/HDA1, SIR2, and HD2 subfamilies. Most *DoHDAC* genes in the same subfamily shared similar structures, and their encoded proteins contained similar motifs, suggesting that the *HDAC* family members are highly conserved and might have similar functions. Different *cis*-acting elements in promoters were related to abiotic stresses and exogenous plant hormones. A transient expression assay in onion epidermal cells by *Agrobacterium*-mediated transformation indicated that all of the detected histone deacetylases such as DoHDA7, DoHDA9, DoHDA10, DoHDT3, DoHDT4, DoSRT1 and DoSRT2, were localized in the nucleus. A tissue-specific analysis based on RNA-seq suggested that *DoHDAC* genes play a role in growth and development in *D. officinale*. The expression profiles of selected *DoHDAC* genes under abiotic stresses and plant hormone treatments were analyzed by qRT-PCR. *DoHDA3*, *DoHDA8*, *DoHDA10* and *DoHDT4* were modulated by multiple abiotic stresses and phytohormones, indicating that these genes were involved in abiotic stress response and phytohormone signaling pathways. These results provide valuable information for molecular studies to further elucidate the function of *DoHDAC* genes.

## Introduction

Higher plants have a sessile lifestyle and often suffer from exposure to abiotic stresses such as drought, cold or high salinity (Shao, Wang & Tang, 2015). Various stress-inducing genes and signaling factors with different functions participate in stress responses, and the expression of these genes usually depends on chromatin remodeling (Kim et al., 2010). The eukaryotic genome is stored in a nuclear protein complex composed of nucleosomes (Teif & Clarkson, 2019). In the nucleosome, two copies of histone H2A, H2B, H3, and H4 form a histone octamer, which is surrounded by DNA 147 base pairs long (Luger et al., 1997). The basic residues such as lysine and arginine in the N-terminal tail of each histone are covalently modified by acetylation to regulate the transcription of genes wrapped around the core histone (Srivastava, Singh & Dubey, 2016). Since a report that indicated that gene activity is related to histone acetylation, the modification of histone acetylation and deacetylation have received increasing attention (Grunstein, 1997). Generally, the acetylation of lysine residues in H3 and H4 N-tails neutralizes the positive charge of the histone tails, thereby reducing their affinity for DNA strands, hence histone acetylation mediated by histone acetyltransferases (HATs), which connect acetyl groups to histones, promotes loosening of the chromatin structure and activates gene transcription (Berger, 2007; Kim et al., 2015; Shahbazian & Grunstein, 2007). Conversely, histone deacetylation mediated by histone deacetylases (HDACs), which remove an acetyl group from histones, facilitates the formation of compact chromatin leading to the repression or silencing of genes (Hollender & Liu, 2008).

HDACs are widespread in eukaryotes from yeast to mammals and plants, and the mechanism by which histone modification leads to the repression of gene expression is conserved (Kim et al., 2015). For example, in *Arabidopsis thaliana*, histone acetylation modification sites were identified by mass-spectrometry and biochemical assays, and were shown to be highly conserved in other eukaryotes (Kim et al., 2015). Based on sequence homology with yeast HDACs, plant HDACs are classified into three different groups, including Reduced Potassium Dependency-3/Histone Deacetylase 1 (RPD3/HDA1), which requires a zinc ion cofactor for deacetylase activity (Yang & Seto, 2007), Silent Information Regulator 2 (SIR2), which depends on nicotinamide adenine dinucleotide (NAD^+^), and Histone Deacetylase 2 (HD2), which is unique in plants (Hirschey, 2011; Pandey et al., 2002). Researchers have identified and characterized some *HDAC* genes in a wide range of plant species, such as maize (*Zea mays*) (Forestan et al., 2018), *A. thaliana* (Hollender & Liu, 2008), rice (*Oryza sativa*) (Fu, Wu & Duan, 2007), barley (*Hordeum vulgare*) (Demetriou et al., 2009), potato (*Solanum chacoense*) (Lagacé et al., 2003), grape (*Vitis vinifera*) (Busconi et al., 2009), tobacco (*Nicotiana tabacum*) (Bourque et al., 2011), black cottonwood (*Populus trichocarpa*) (Alinsug, Yu & Wu, 2009), banana (*Musa acuminata*) (Fu et al., 2018), soybean (*Glycine max*) (Yang et al., 2018), litchi (*Litchi chinensis*) (Peng et al., 2017), common bean (*Phaseolus vulgaris*) (Hayford et al., 2017), common liverwort (*Marchantia polymorpha*) (Chu & Chen, 2018) and tomato (*Solanum lycopersicum*) (Zhao et al., 2015), and have studied the functions of certain *HDAC* genes. For example, *HDAC* genes are involved in plant responses to stress-related hormones such as salicylic acid (SA), abscisic acid (ABA) or jasmonic acid (JA) and stress stimuli such as drought, cold, salt, and pathogens (Ma et al., 2013). *AtHDA6* and *AtHDA19* regulate gene expression induced by ABA and salt stress in *A. thaliana* (Chen & Wu, 2010). Overexpression of *AtHD2C* made transgenic *A. thaliana* plants insensitive to ABA and tolerant to salt and drought stresses (Sridha & Wu, 2006). However, the molecular functions of many of these HDAC proteins, which are histone modifiers, have not yet been well characterized.

*Dendrobium* Swartz is the second largest genus of the Orchidaceae, which consists of nearly 1,600 accepted species (Juswara, Schuiteman & Champion, 2019). Among them, *Dendrobium officinale* Kimura et Migo is a popular tonic and traditional Chinese medicine (TCM) with high commercial value, containing compounds with antioxidant and antitumor activity (Li et al., 2011; Teixeira da Silva & Ng, 2017; Wu et al., 2014). In addition to polysaccharides thought to be among the major active ingredients of *D. officinale*, a wide variety of low molecular weight compounds including bibenzyls, phenanthrenes, and flavanones have been detected by phytochemical analyses (Chen et al., 2012). *D. officinale* grows well under a suitable environment (Ding et al., 2018; Yang et al., 2014). In a natural environment, *D. officinale* is in an epiphytic state, often growing on humid rocks in mountain climates at an altitude of 500–1600 m, or on the tree trunks in virgin forests with warm and moist environments (Zeng et al., 2019). Based on its growth habits, *D. officinale* is susceptible to abiotic stresses such as high and low temperatures, drought and salinization, resulting in low natural reproduction rate and slow growth (Zeng et al., 2019).

Phytohormones not only mediate developmental processes in plants but can also sustainably alleviate adverse effects of abiotic and biotic stresses in plants (Wolters & Jurgens, 2009). For instance, SA improved plant abiotic stress tolerance via SA-mediated control of major plant metabolic processes by strengthening salinity and drought stress tolerance (Khan et al., 2015). The JA signaling pathway plays a key role in coordinating the activation of biosynthetic routes involved in defense-related metabolites (Ballare & Austin, 2019). Secondary metabolites, including terpenes, phenolics, compounds with nitrogen, and others, which are biosynthetically derived from primary metabolites, have also been established as beneficial for plants’ tolerance to biotic and abiotic stresses (Ramakrishna & Ravishankar, 2011). Environmental stresses trigger the biosynthesis of certain secondary metabolites in plants which are important candidates for human nutrition (Ashraf et al., 2018). Furthermore, epigenetic regulation of gene expression is important for a plant’s adaptation to environmental changes (Zheng et al., 2020). Therefore, understanding how histone acetylation modification is involved in the responses of *D. officinale* to environmental stresses will contribute significantly to our understanding of the molecular mechanisms underlying epigenetic regulation in this orchid. However, studies on the evolutionary relationships and functional characteristics of *HDAC* genes in *D. officinale* (*DoHDAC* genes) are scarce.

The genome and transcriptome sequences of *D. officinale* are now available, allowing the *DoHDAC* genes to be isolated and identified (Shen et al., 2017). In this study, fourteen *DoHDAC* genes were identified from the *D. officinale* genome and their structure, phylogeny, conserved motifs, and putative promoter were analyzed. Subsequently, the subcellular localization and expression patterns of selected *DoHDAC* genes under hormone, salt, drought and cold treatments were analyzed. These results provide a foundation for further clarifying the functions of DoHDAC proteins.

## Material and Methods

### Identification and bioinformatics analysis of *DoHDAC* genes

To identify potential *DoHDAC* genes, the coding sequences (CDSs) of *AtHDAC* genes were used for a BLASTP algorithm-based query against the *D. officinale* genome database (https://www.ncbi.nlm.nih.gov/genome/?term=Dendrobium, NCBI Biosample ID: SAMN03083363). Genes were identified by a hidden Markov model search based on the HDAC domain using the Pfam protein domain database. To determine the sequence identity among HDACs, full-length amino acid sequences of HDAC proteins were aligned and compared using *DNAMAN 8* software (Lynnon Biosoft, San Ramon, CA, USA). For phylogenetic analysis, the amino acid sequences of HDAC proteins from *D. officinale*, *A*. *thaliana* and *O*. *sativa* in *FASTA* format were aligned with Clustal X 2.0 (Larkin et al., 2007), and sequence identity was calculated by UniProt BLAST (http://www.uniprot.org/blast/), and then a neighbor-joining phylogenetic tree was constructed using the *MEGA 7* program (Kumar, Stecher & Tamura, 2016) with a bootstrap analysis of 1000 replicates. Nucleus localization signals (NLSs) were found in amino acid sequences of DoHDAC proteins at http://localizer.csiro.au/ (Sperschneider et al., 2017). Recognizable conserved domains of DoHDAC proteins were identified with EBL-EBI (http://www.ebi.ac.uk/interpro/search/sequence-search) and also verified by the CDD database (https://www.ncbi.nlm.nih.gov/cdd). The domain architecture was drawn using DOG2.0 software (Ren et al., 2009). The protein sequence motifs of DoHDAC proteins were identified using MEME (http://memesuite.org/tools/meme) (Bailey et al., 2009). Gene structure analysis of the *DoHDAC* genes was conducted with GSDS (Gene Structure Display Server, http://gsds.gao-lab.org/) (Hu et al., 2014). The functional interacting networks of functional proteins were integrated in STRING (https://string-db.org/) based on an *A. thaliana* association model with the confidence parameter set at a threshold of 0.700 and no more than 20 interactors (Szklarczyk et al., 2019). DNA sequences about 2000 bp long upstream of the initiation codon ATG of *DoHDAC* genes were regarded as putative promoters. *Cis*-elements in these promoters were analyzed using the online program PlantCare (http://bioinformatics.psb.ugent.be/webtools/plantcare/html/). The predicted *cis*-acting elements of promoters of *DoHDAC* genes were illustrated by TBtools software (Chen et al., 2020).

### Subcellular localization assays

The CDSs of *DoHDA7*, *DoHDA9*, *DoHDA10*, *DoHDT3*, *DoHDT4*, *DoSRT1* and *DoSRT2* without a termination codon were cloned into binary vector pCAMBIA1302 with the green fluorescent protein (GFP) (GenBank Accession No. AF234298) with the *Nco* I restriction enzyme. The resulting binary vectors, pCAMBIA1302-*DoHDA7*, pCAMBIA1302-*DoHDA9*, pCAMBIA1302-*DoHDA10*, pCAMBIA1302-*DoHDT3*, pCAMBIA1302-*DoHDT4*, pCAMBIA1302-*DoSRT1* and pCAMBIA1302-*DoSRT2*, were transferred into *Agrobacterium tumefaciens* strain EHA105 (Shanghai Weidi Biotechnology Co. Ltd., Shanghai, China), according to the manufacturer’s protocol. Then, using *Agrobacterium*-mediated transformation, these recombinant binary vectors were transformed into epidermal cells of onion (*Allium cepa* L. “Red Sun”) (Huang et al., 2011). A Zeiss Model Axio Imager A2 upright fluorescence microscope (Carl Zeiss, Oberkochen, Germany) was used to observe gene expression in transformed onion epidermal cells. In order to show the nucleus, according to the manufacturer’s protocol, the epidermal cell layers were stained with 2-(4-amiylphenyl)-6-indolecarbamoyl dihydrochloride (DAPI) staining solution (Beyotime Biotech Inc., Shanghai, China). The positive control was onion epidermal cells with an empty pCAMBIA1302-GFP vector. ZEN 2011 software (Carl Zeiss) was used to analyze cellular localization.

### Plant materials and treatments

Based on previously reported concentrations (He et al., 2017), *D. officinale* plantlets with a stem length of about three cm growing on half-strength Murashige and Skoog (MS; (Murashige & Skoog, 1962) (1/2 MS) agar medium containing 2% sucrose were separately exposed to one of several abiotic stresses: 100 *μ*M gibberellic acid (GA_3_), 100 *μ*M ABA, 100 *μ*M SA, 200 *μ*M methyl jasmonate (MeJA), 15% polyethylene glycol (PEG)-6000 (all compounds: Sigma-Aldrich, Shanghai, China), 250 mM NaCl (Guangzhou Chemical Reagent Factory, Guangzhou, China) and 4 °C , separately. Five *D. officinale* plantlets were used for each treatment. Plant tissues, including roots, stems and leaves, were harvested at different selected time points after applying the above treatments, then immediately frozen in liquid nitrogen for further gene expression experiments. Based on the transcriptome data, the expression patterns of *DoHDAC* genes in different tissues were analyzed, and a heatmap was generated with the heatmap software package on the BMKCloud platform (http://www.biocloud.net).

About 100 mg of samples, including leaves, roots and stems from previously collected *D. officinale* plants, were placed in a pre-chilled mortar and ground with liquid nitrogen to a fine powder. Each finely powdered sample was transferred to a 2 mL microcentrifuge tube containing 1.5 mL of pre-warmed extraction buffer (50 mM Tris–HCl, 20 mM EDTA, 1 M NaCl, 2% SDS and 4% β-mercaptoethanol; pH 8.0). Samples were incubated in a water bath at 65 °C for 15–20 min. The tube was centrifuged at 13,000 g for 10 min at 4 °C and the supernatant was transferred to a sterile 2 mL centrifuge tube. An equal volume of water-saturated phenol (pH 4.5) was added, vortexed for 2 min, then kept at room temperature for 10 min. The tube was centrifuged at 13,000 g for 10 min at 4 ° C, the supernatant was transferred to a sterile 2 mL centrifuge tube, then equal volumes of chloroform and isoamyl alcohol (24/1, v/v) were added and mixed thoroughly. The tube was again centrifuged at 13,000 g for 10 min at 4 °C, the supernatant was transferred to a sterile two mL centrifuge tube, and an equal volume of isopropanol was added. The solution in the centrifuge tube was mixed, placed in a refrigerator at −20 °C for 10 min, and then centrifuged at 13,000 g for 10 min at 4 °C. The supernatant was discarded, the pellet was washed with 75% chilled ethanol, and the tube was centrifuged at 13,000 g for 15 min at 4 °C. The RNA pellet was dried at room temperature for 5 min. The dried RNA pellet was resuspended in 50 *μ*L of diethyl phosphorocyanidate (DEPC) in distilled water. According to the manufacturer’s instructions, samples were treated with Recombinant DNase I (RNase-free) (Takara Bio Inc., Kusatsu, Shiga, Japan) to remove genomic DNA, and stored at −80 °C. The NanoDrop One/OneC micro nucleic acid protein concentration analyzer (Thermo Fisher Scientific, Waltham, MA, USA) was used to assess the purity and concentration of RNA samples. Agarose gel electrophoresis was used to survey the RNA integrity by Clinx GenoSens gel documentation system (Clinx Science Instruments, Shanghai, China).

### Quantitative real-time reverse transcriptase-PCR analysis

Following the manufacturer’s instructions, the first-strand cDNA was synthesized using the GoScript™ Reverse Transcription System (Promega, Madison, WI, USA). Quantitative real-time reverse transcriptase-PCR (qRT-PCR) assays of three biological replicate samples were performed on a LightCycler 480 system (Roche, Basel, Switzerland) using the Aptamer™ qPCR SYBR^^®^^ Green Master Mix (Tianjin Novogene Bioinformatics Technology Co. Ltd., Tianjin, China). The reaction conditions were as follows: 95 °C for 5 min, followed by 40 cycles of 95 °C for 10 s, and 60 °C for 30 s. *D. officinale* actin (NCBI accession number JX294908) was used as an internal control gene based on the advice of He et al. (2015). The relative expression levels of genes were calculated using the 2^−ΔΔ*CT*^ method (Livak & Schmittgen, 2001). The expression of genes was significantly altered upon different treatments when the fold change was greater than 2 (Li et al., 2016). The gene-specific primers for qRT-PCR were designed by Primerquest (https://sg.idtdna.com/Primerquest/Home/Index) and are listed in Table S1.

## Results

### Identification and phylogenetic analysis of the *DoHDAC* genes

**Figure 1:**
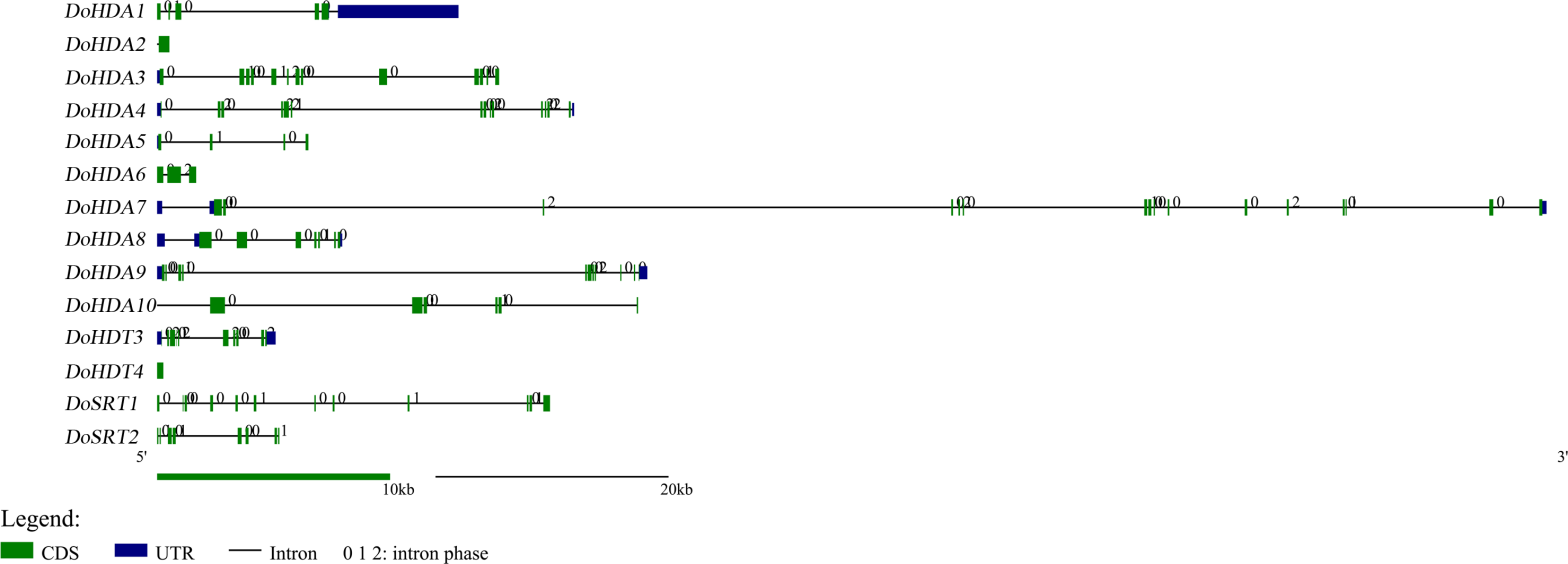
Exon-intron structures of *DoHDAC* genes. Untranslated regions, exons and introns are indicated by blue, green and black, respectively. The green scale bar represents 10 kb. The black scale line represents 20 kb.

**Figure 2:**
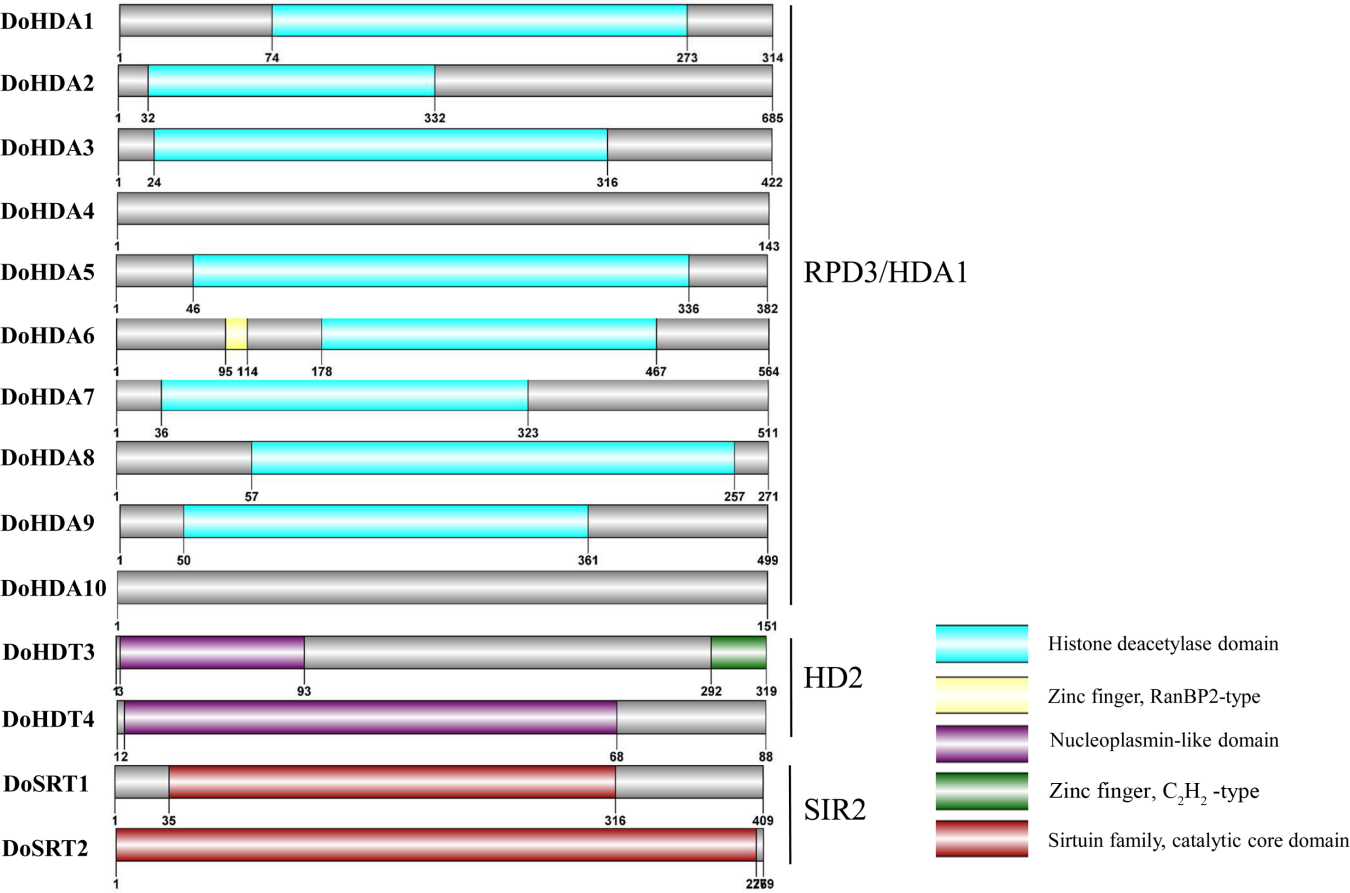
Conserved domain of *DoHDAC* genes. The location and size of domains are shown by different colors. Proteins belonging to each family are grouped together.

**Figure 3:**
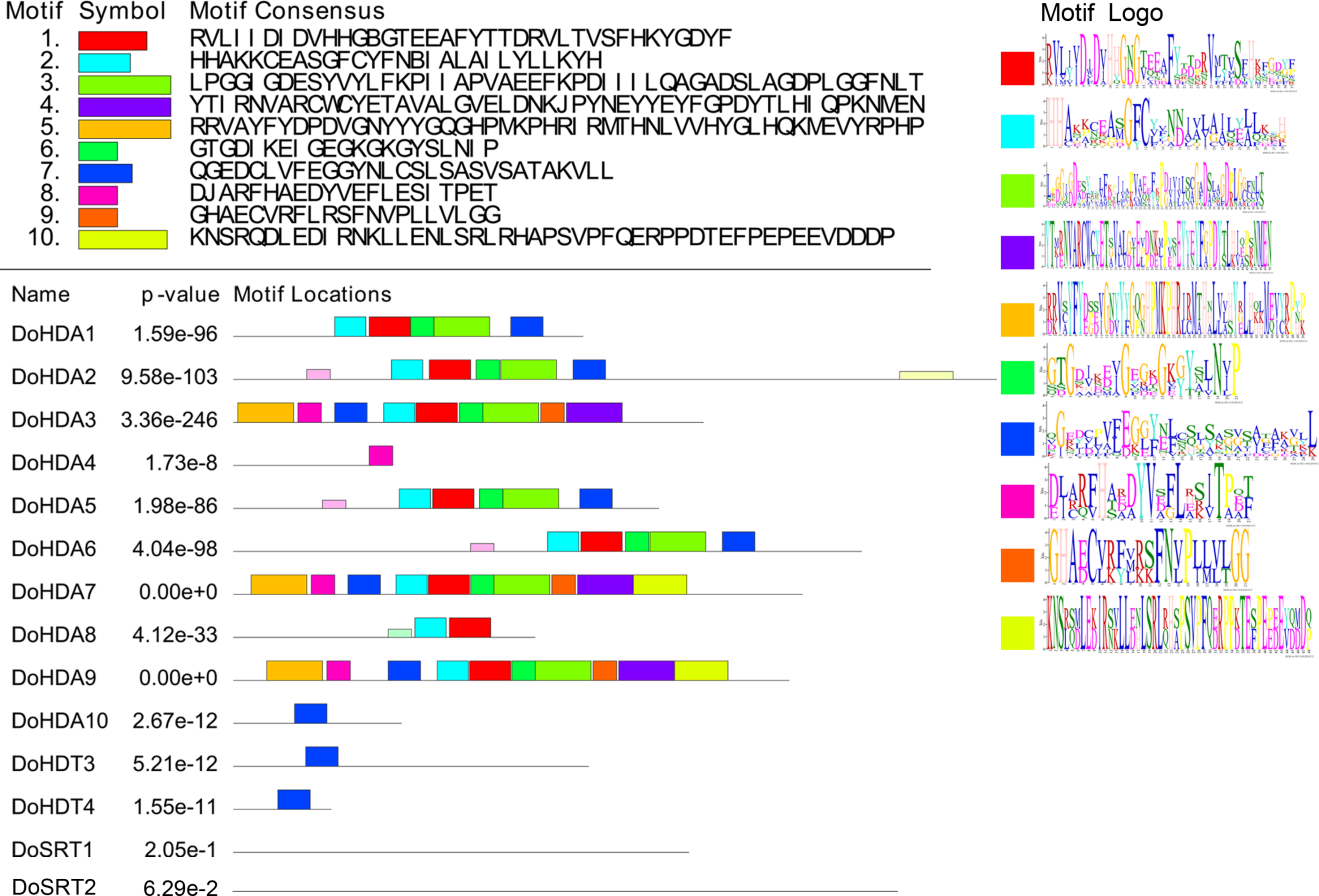
Conserved motifs analysis of the DoHDAC proteins. The conserved protein sequence motifs of the DoHDAC proteins were identified using the MEME program. Each motif is represented by a different color.

**Figure 4:**
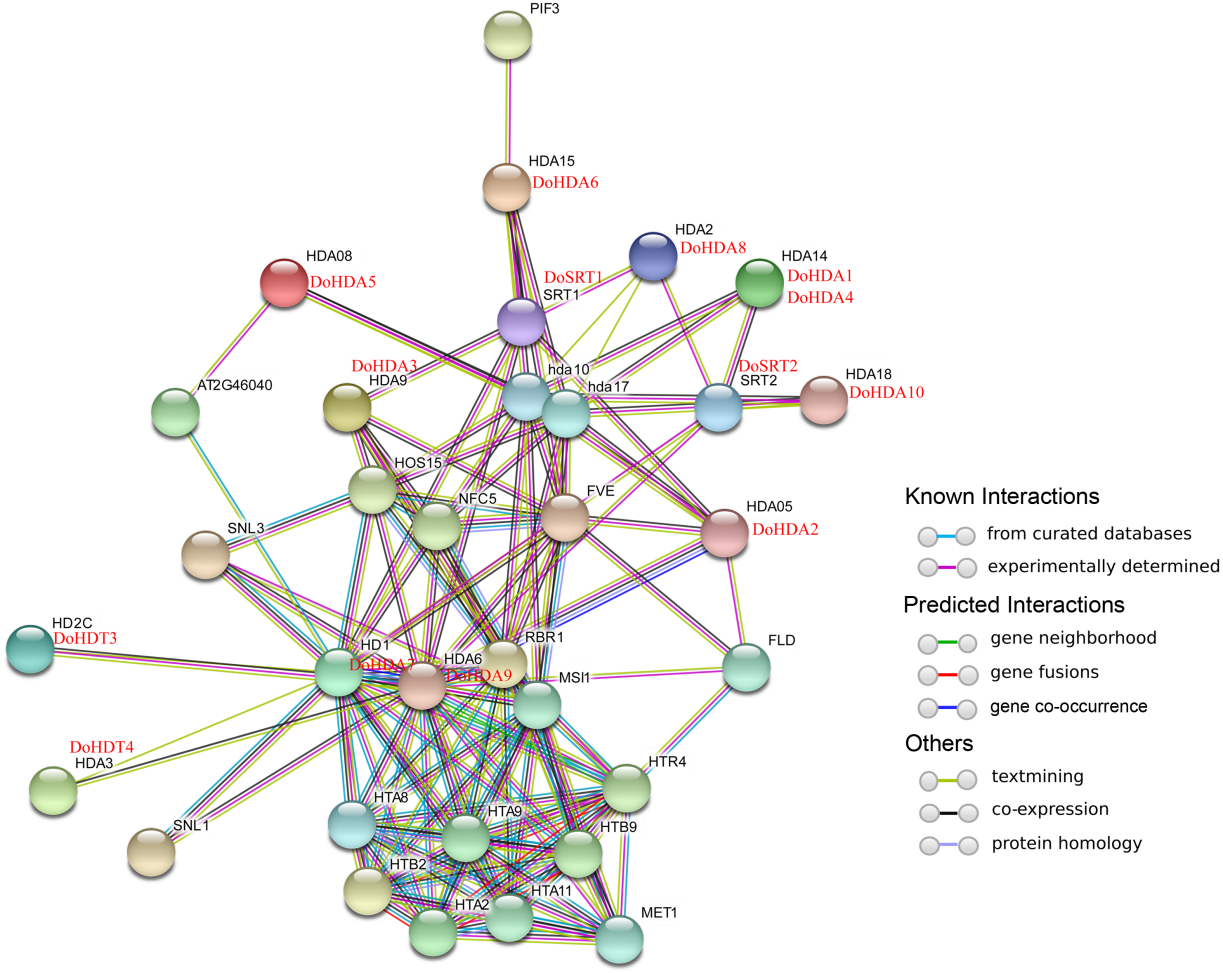
Interaction networks of DoHDAC proteins** according to the orthologs in *A. thaliana*. Different colored lines represent types of evidence for the association.

**Figure 5:**
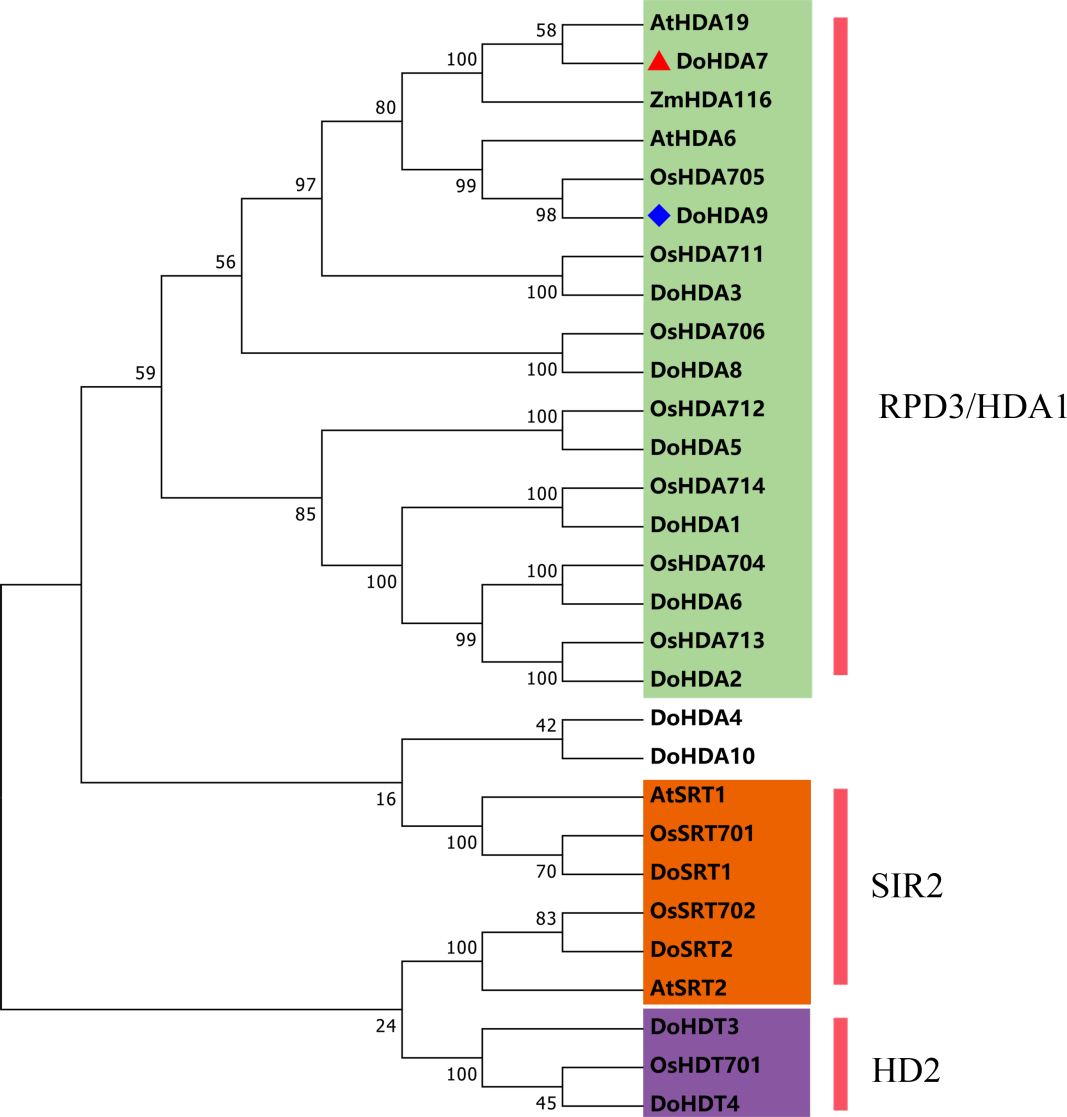
Phylogenetic tree of the deduced amino acid sequences of HDAC proteins in *D . officinale*, *O. sativa*, *A. thaliana*, and *Z. mays*. Abbreviations for species are as follows: *D. officinale* (Do), *A. thaliana* (At), *O. sativa* (Os),** and *Z. mays* (Zm). Enzymes used for alignment were as follows: AtHDA6, AT5G63110; AtHDA19, AT4G38130; AtSRT1, AT5G55760; AtSRT2, AT5G09230; OsHDA704, Os07g0164100; OsHDA705, Os08g0344100; OsHDA706, Os06g0571100; OsHDA711, Os04g0409600; OsHDA712, Os05g0440100; OsHDA713, Os07g0602200; OsHDA714, Os12g0182700; OsHDT701, Os05g0597100; OsSRT701, Os04g0271000; OsSRT702, Os12g0179800, ZmHDA116, Zm00001d046388.

In this study, 14 *DoHDAC* genes were identified in the *D. officinale* genome. The CDS length of *DoHDAC* genes was diverse, ranging from 267 bp to 2085 bp (Table 1). Analysis of gene structure helps to understand gene function and evolution (Feng et al., 2016). Hence, the structure of the *DoHDAC* genes was analyzed. Our results showed that their conserved coding regions consisted of different numbers of exons, while *DoHDA10* and *DoHDT4* have only one exon (Fig. 1). Amino acid sequence alignment of the 14 DoHDAC proteins showed that, except for DoHDA4 and DoHDA10, the eight histone deacetylases with amino acid sequences between 200 aa and 312 aa all contained a typical histone deacetylase catalytic domain. DoHDA4 and DoHDA10 showed significantly low sequence identity with other RPD3/HDA1 family proteins in *D. officinale* (Fig. 2), suggesting that they might play some unique roles in certain cellular events. Functional domain analysis revealed that DoHDA6 possessed a RanBP2-type zinc finger domain (Fig. 2). DoHDT3 contained a conserved domain with a pentapeptide (MEFWG) on the N-terminus and a C_2_H_2_ zinc finger domain on the C-terminus of the protein (Fig. S1, Fig. 2). In addition, DoSRT1 and DoSRT2 possessed a single copy of the sirtuin-type HDAC domain (Fig. 2). To investigate the conserved nature of motifs in DoHDAC proteins, we investigated 10 motifs among the 14 DoHDAC proteins. The width of these 10 motifs ranged from 21 (motif 6, 8 and 9) to 50 (motif 3, 4 and 5) amino acids (Fig. 3). Closely related proteins, such as DoHDA3, DoHDA7, DoHDA9, DoHDA1, DoHDA6, DoHDA2, DoHDA5, DoHDT3 and DoHDT4, had similar conserved motifs. For instance, most of the *D. officinale* RPD3/HDA1 superfamily proteins contained HDACs domain motifs 1 and 2. However, the HDAC domain motif was not found in DoSRT1 and DoSRT2. We constructed a protein–protein interaction network of the 14 DoHDAC proteins using STRING (http://string-db.org/) based on an *A*. *thaliana* association model (Fig. 4). DoHDA9 showed a high degree of amino acid sequence similarity to AtHDA6, thus it might interact with the flavin-containing amine oxidoreductase family protein (FLD), which might be histone demethylase that promotes flowering by repressing FLOWERING LOCUS C (FLC) (Table S3, Fig. 4, Table S4). Moreover, DoHDA9 might interact with the WD40 repeat-containing protein HOS15 in response to abiotic stress (Table S4). To study the evolutionary relationships among DoHDAC proteins, a phylogenetic tree was constructed by aligning the amino acid sequences of histone deacetylases of four, ten and one HDAC proteins from *A. thaliana*, *O. sativa*, and *Z. mays* using MEGA 7.0. In the phylogenetic tree, 14 DoHDAC proteins were divided into three families: RPD3/HDA1, SIR2, and HD2 (Fig. 5). DoHDA9, AtHDA6 homologue, shared approximately 71.1% amino acid sequence similarity with AtHDA6. In addition, DoHDA7, AtHDA19 homologue, had approximately 80% amino acid sequence similarity with AtHDA19.

**Table 1:** Overview of histone deacetylase genes identified in *D. officinale*.

Gene	CDS length (bp)	Protein attributes		
		Length (aa)	MW (Da)^*^	pI^*^
*DoHDA1*	945	314	34352.98	6.49
*DoHDA2*	2058	685	76108.86	5.06
*DoHDA3*	1269	422	105199.42	5.06
*DoHDA4*	432	143	15457.30	5.69
*DoHDA5*	1149	382	41545.88	5.44
*DoHDA6*	1695	564	62307.71	6.31
*DoHDA7*	1536	511	57896.33	5.30
*DoHDA8*	816	271	30616.14	6.25
*DoHDA9*	1500	499	55436.99	5.45
*DoHDA10*	456	151	16728.54	6.27
*DoHDT3*	960	319	76918.49	5.15
*DoHDT4*	267	88	10000.50	6.25
*DoSRT1*	1230	409	45501.65	8.19
*DoSRS2*	840	279	30821.09	7.04

**Notes.**

* The theoretical isoelectric point (pI) and molecular weight (Mw) of the *D. officinale* HDAC proteins were calculated with the Compute pI/Mw online tool (https://web.expasy.org/compute_pi/).

### Subcellular localization analysis of selected *DoHDAC* genes

**Figure 6:**
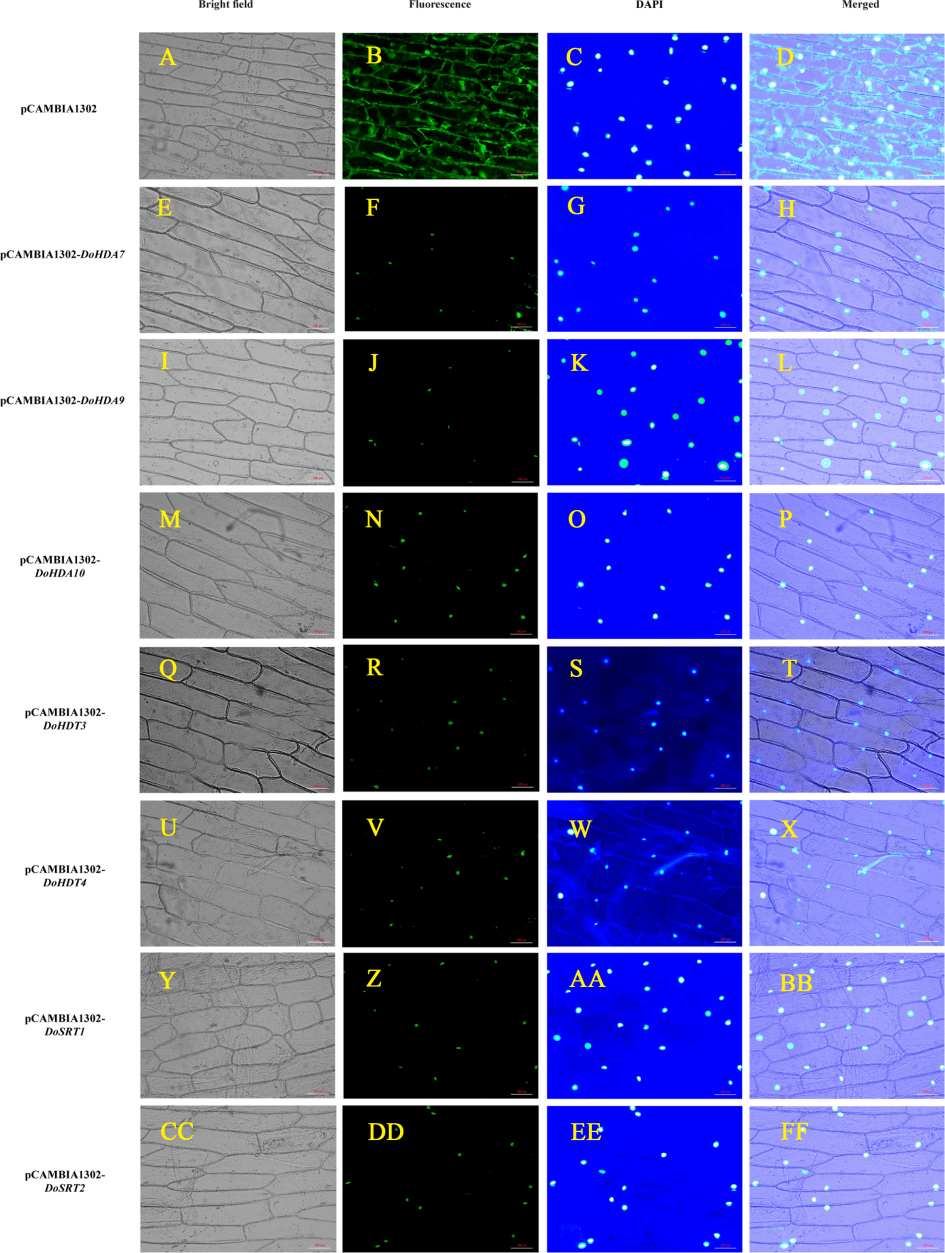
Subcellular localization analysis of DoHDAC proteins. pCAMBIA1302-*DoHDA7*, pCAMBIA1302-*DoHDA9*, pCAMBIA1302-*DoHDA10*, pCAMBIA1302-*DoHDT3*, pCAMBIA1302-*DoHDT4*, pCAMBIA1302-*DoSRT1* and pCAMBIA1302-*DoSRT2* was inserted into onion epidermal cells by *Agrobacterium*-mediated transformation to determine their subcellular localization. (A–D) GFP fluorescence distributed throughout the entire cells from the GFP empty vector. (E–H) GFP fluorescence from cells expressing DoHDA7-GFP fusion protein localized to the nucleolus. (I–L) GFP fluorescence from cells expressing DoHDA9-GFP fusion protein localized to the nucleolus. (M–P) GFP fluorescence from cells expressing DoHDA10-GFP fusion protein localized to the nucleolus. (Q–T) GFP fluorescence from cells expressing DoHDT3-GFP fusion protein localized to the nucleolus. (U–X) GFP fluorescence from cells expressing DoHDT4-GFP fusion protein localized to the nucleolus. (Y–BB) GFP fluorescence from cells expressing DoSRT1-GFP fusion protein localized to the nucleolus. (CC–FF) GFP fluorescence from cells expressing DoSRT2-GFP fusion protein localized to the nucleolus. The panels from left to right correspond to brightfield, fluorescence (GFP), DAPI staining images and the merged images of fluorescence and brightfield, respectively. Scale bars: 100 µm.

To verify the subcellular localization of certain DoHDAC proteins, an *Agrobacterium*-mediated transient transformation system was used in onion epidermal cells. The expression of *GFP* fused to *DoHDA7*, *DoHDA9*, *DoHDA10*, *DoHDT3*, *DoHDT4*, *DoSRT1* and *DoSRT2* was tracked by the GFP marker signal. The blue fluorescence pattern of DAPI-stained cells completely merged with the green fluorescence pattern, showing the nuclear localization of DoHDAC proteins in onion epidermal cells (Fig. 6). As shown in Fig. 6, the positive control was distributed throughout the onion cells. In contrast, DoHDA7, DoHDA9, DoHDA10, DoHDT3, DoHDT4, DoSRT1 and DoSRT2 were localized in the nucleus.

### Analysis of *cis*-acting elements in the putative promoters of *DoHDAC* genes

**Figure 7:**
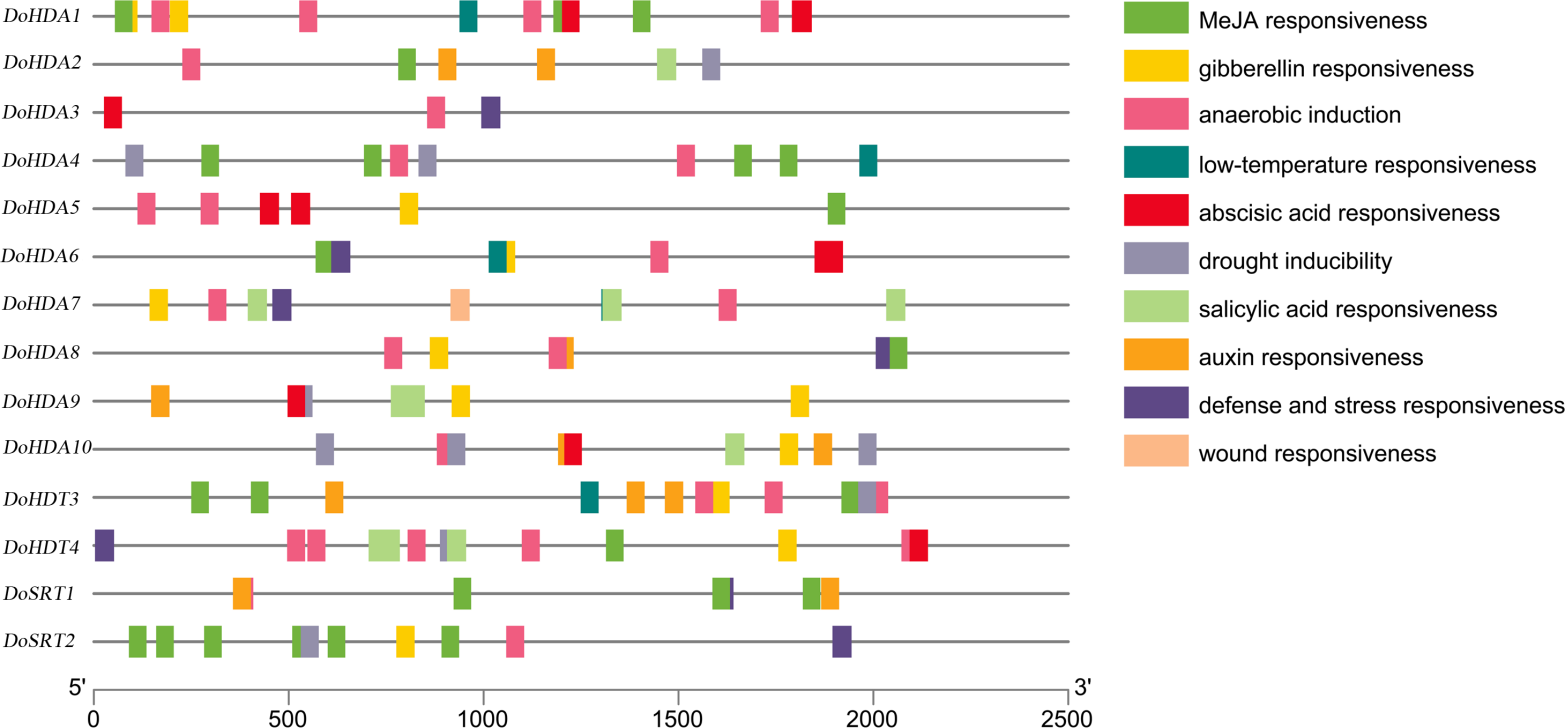
About 2 kb upstream sequences of *DoHDAC* genes and distribution of predicted *cis*-elements. Different *cis*-elements are represented using different colored boxes.

A *cis*-acting element is a non-coding part of DNA, is usually limited to the 5′ upstream region of a gene, and is responsible for transcriptional regulation. To better predict gene functions, we identified the *cis*-acting elements within the putative ∼2 kb promoter region of *DoHDAC* genes. In addition to the core *cis*-acting elements, such as the TATA-box and the CAAT-box, several other *cis*-elements related to plant growth or development, stress responses, and hormone responses were detected (Table S2, Fig. 7). *Cis*-acting elements related to growth or development included light-responsive elements such as Box4, G-box, GT1-motif, Sp1, and TCT-motif, elements expressed only in the endosperm such as the AACA_motif and GCN4_motif, and elements under circadian control or showing meristem-specific activation such as CAT-box and NON-box. *Cis*-acting elements related to phytohormone responses included GA-responsive elements such as GARE-motif, P-box and TATC-box, MeJA-responsive elements such as CGTCA-motif and TGACG-motif, ABA-responsive element (ABRE), and SA-responsive element such as TCA-element. *Cis*-acting elements related to abiotic stresses included drought-responsive elements such as MBS and a low-temperature-responsive (LTR) element. For example, both *DoHDA3* and *DoHDT3* had *cis*-acting elements involved in MeJA-responsiveness, low-temperature responsiveness and drought inducibility. Therefore, based on the large number of *cis*-acting elements related to these stresses, the *D. officinale* plantlets were treated with SA, MeJA, ABA, GA_3_, cold stress and drought stress.

### Expression analysis of *DoHDAC* genes in different tissues

**Figure 8:**
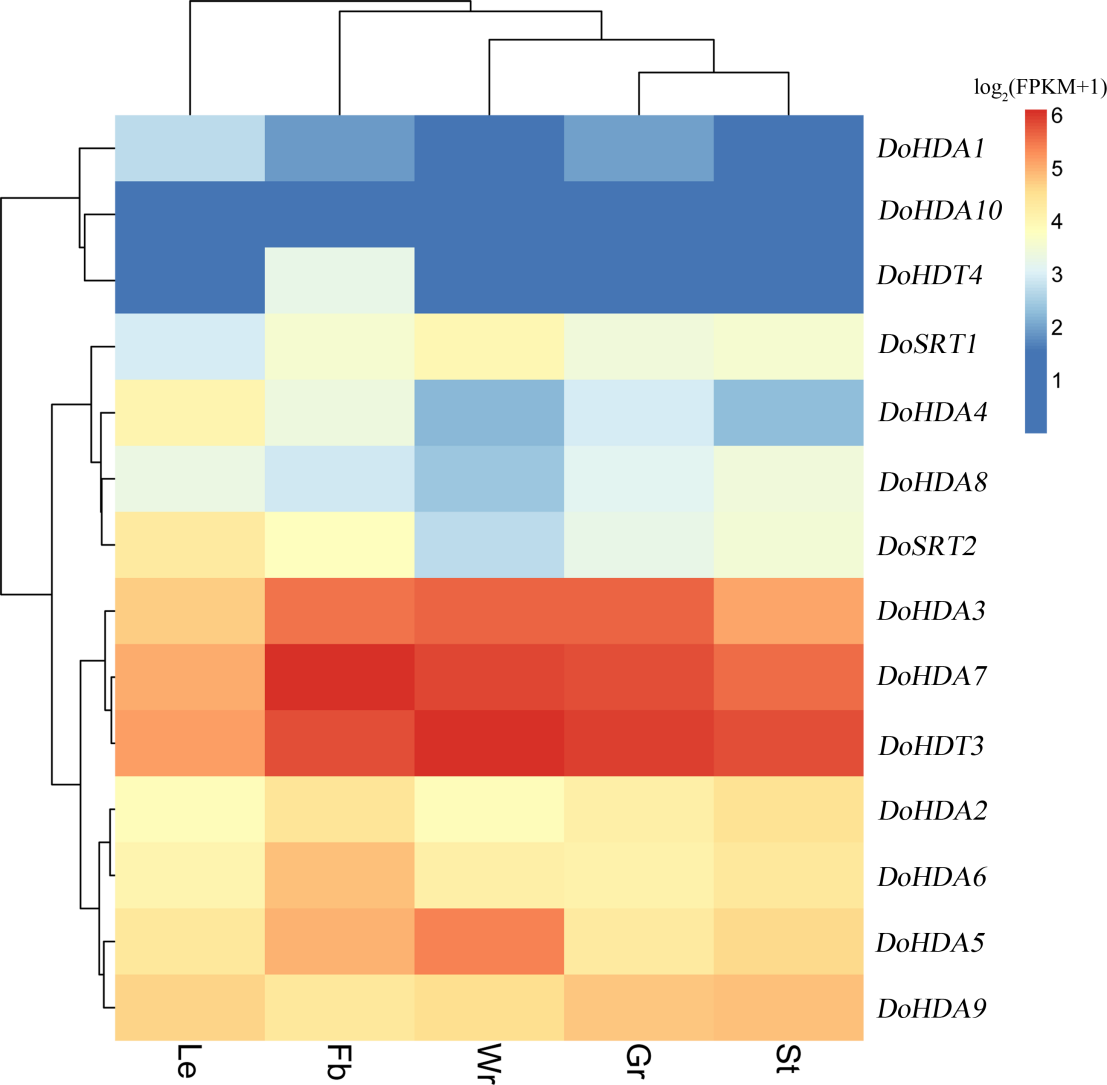
Expression profiles of *DoHDAC* genes in different tissues based on the transcriptome data. These data were used to analyze the expression profiles of *DoHDAC* genes in five different tissues: the white part of root (Wr), green root tip (Gr), stem (St), leaf (Le), and flower bud (Fb). The color bar represents the log_2_(FKPM+1) values of each gene and transcript in different tissues. Red represents high relative expression, and blue represents low relative expression.

Since high-throughput sequencing has been performed on *D. officinale* tissues at different developmental stages, the publicly available RNA-seq data obtained is a useful resource for studying gene expression profiles. We used previously reported transcriptome data (Zhang et al., 2017) to examine the expression pattern of *DoHDAC* genes in different tissues. The heat map (Fig. 8) revealed that three *DoHDAC* genes (*DoHDA3*, *DoHDA7*, and *DoHDT3*) were highly expressed with FPKM values > 25 in all detected tissues, while *DoHDA10* showed a low expression with FPKM values < 5 in all the detected tissues. In addition, some genes displayed a tissue-specific expression pattern. For example, *DoHDA5* and *DoSRT1* were most highly expressed in the white parts of root, while *DoHDA6* and *DoHDT4* were expressed predominantly in flower buds (Fig. 8). These results suggested that *DoHDAC* genes might play an important role in the development of different organs.

### Expression analysis of *DoHDAC* genes in response to exogenous phytohormones

**Figure 9:**
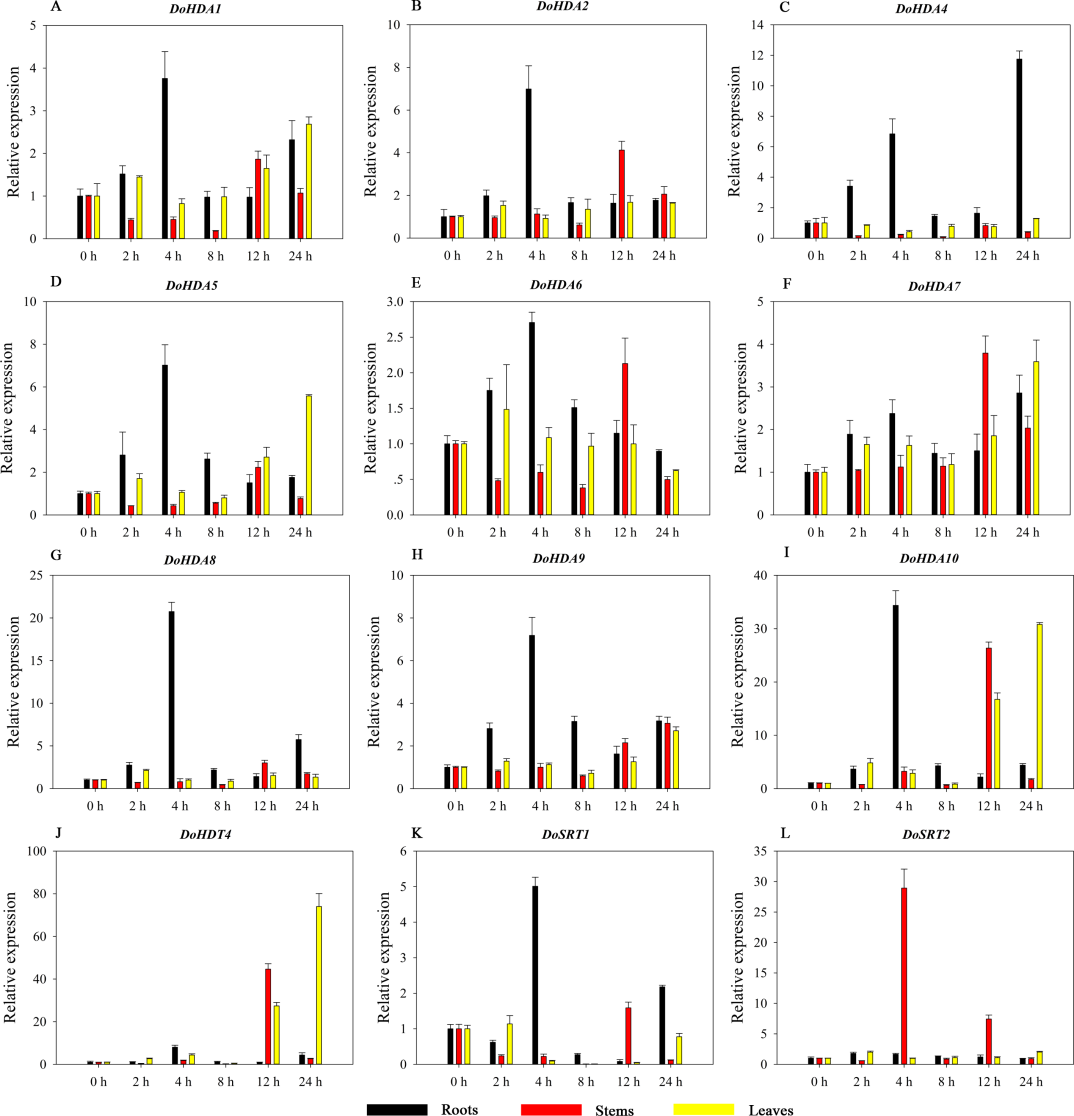
Quantitative real-time reverse transcriptase-PCR analysis of expression of selected *DoHDAC* genes at different hours of GA_3_ treatment in different tissues. A–L represent *DoHDA1*, *DoHDA2*, *DoHDA4*, *DoHDA5*, *DoHDA6*, *DoHDA7*, *DoHDA8*, *DoHDA9*, *DoHDA10*, *DoHDT4*, *DoSRT1*, *DoSRT2*, respectively. Error bars represent the standard error of the means of three independent replicates. Expression levels were calculated relative to 0 h of each organ.

**Figure 10:**
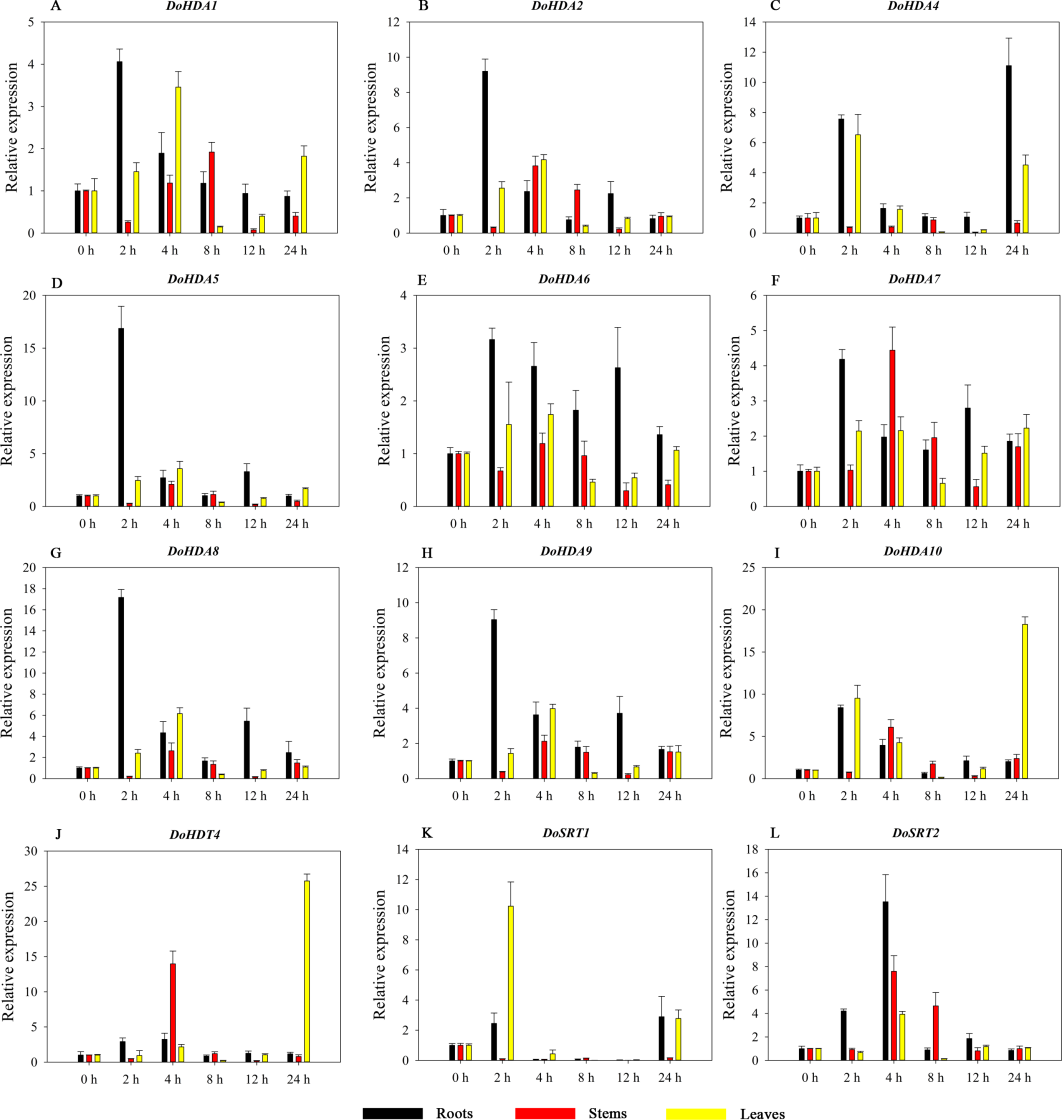
Quantitative real-time reverse transcriptase-PCR analysis of expression of selected *DoHDAC* genes at different hours of SA treatment in different tissues. A–L represent *DoHDA1*, *DoHDA2*, *DoHDA4*, *DoHDA5*, *DoHDA6*, *DoHDA7*, *DoHDA8*, *DoHDA9*, *DoHDA10*, *DoHDT4*, *DoSRT1*, *DoSRT2*, respectively. Error bars represent the standard error of the means of three independent replicates. Expression levels were calculated relative to 0 h of each organ.

**Figure 11:**
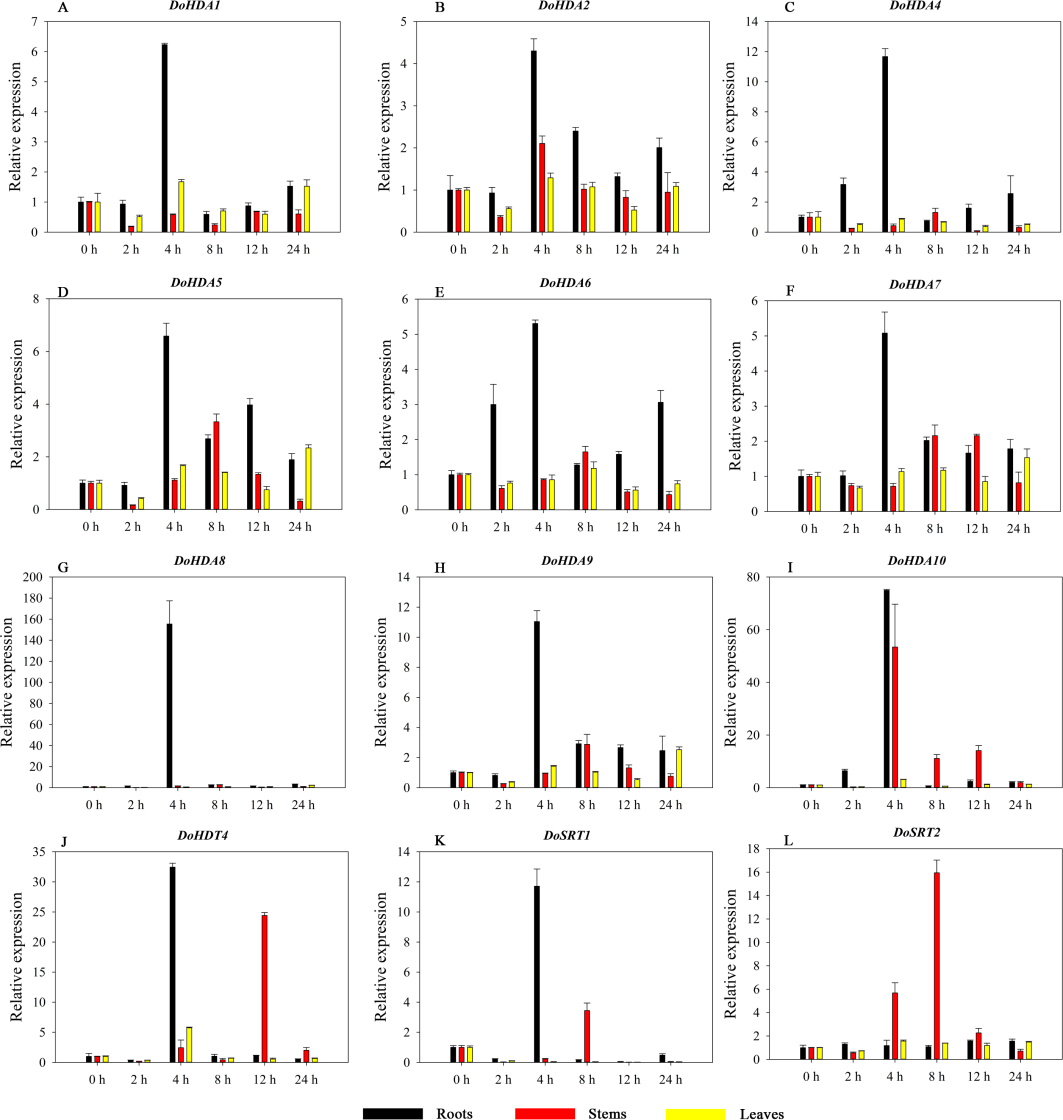
Quantitative real-time reverse transcriptase-PCR analysis of expression of selected *DoHDAC* genes at different hours of ABA treatment in different tissues. A–L represent *DoHDA1*, *DoHDA2*, *DoHDA4*, *DoHDA5*, *DoHDA6*, *DoHDA7*, *DoHDA8*, *DoHDA9*, *DoHDA10*, *DoHDT4*, *DoSRT1*, *DoSRT2*, respectively. Error bars represent the standard error of the means of three independent replicates. Expression levels were calculated relative to 0 h of each organ.

**Figure 12:**
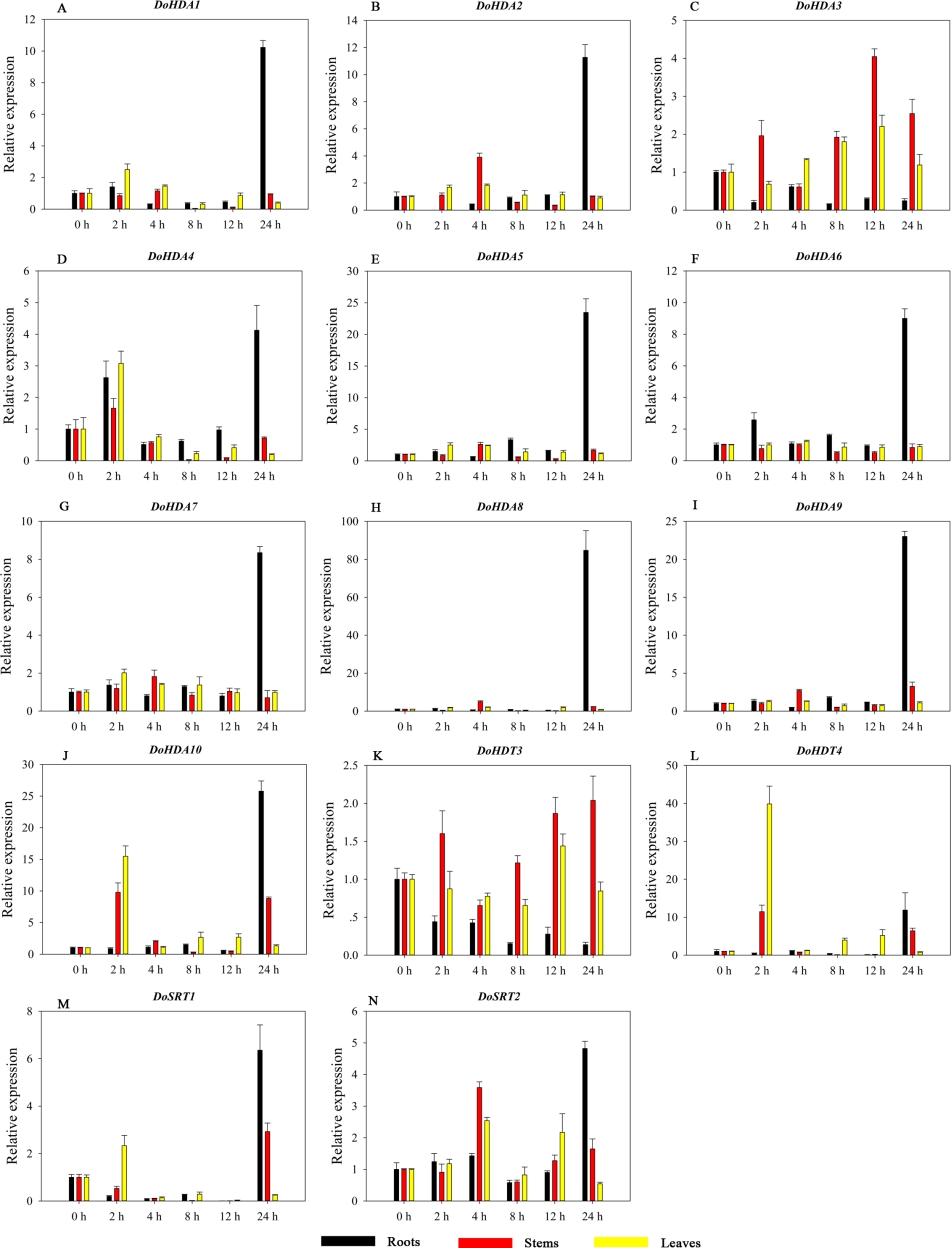
Quantitative real-time reverse transcriptase-PCR analysis of expression of selected *DoHDAC* genes at different hours of MeJA treatment in different tissues. A–N represent *DoHDA1*, *DoHDA2*, *DoHDA3*, *DoHDA4*, *DoHDA5*, *DoHDA6*, *DoHDA7*, *DoHDA8*, *DoHDA9*, *DoHDA10*, *DoHDT3*, *DoHDT4*, *DoSRT1*, *DoSRT2*, respectively. Error bars represent the standard error of the means of three independent replicates. Expression levels were calculated relative to 0 h of each organ.

Hormones play a vital role in the vegetative and reproductive growth of plants. Analysis of the *cis*-acting elements in promoters indicates that the *DoHDAC* genes might respond to a variety of stresses and signaling molecules. Therefore, based on potential *cis*-acting elements, we selected some genes and used qRT-PCR to detect their expression patterns under various phytohormone treatments. We analyzed the expression patterns of 12 *DoHDAC* genes in different subfamilies after GA_3_ treatment (Fig. 9). The results showed that after 4 h of GA_3_ treatment, 11 genes in roots were highly up-regulated, *DoHDA8* and *DoHDA10* were the highest (> 20-fold), and *DoSRT2* was highly up-regulated in stems. In addition, after 24 h of GA_3_ treatment, *DoHDA10* and *DoHDT4* were highly up-regulated in leaves. At the same time, *cis*-acting elements involved in the GA_3_-response were found in these four genes (Table S2). Most selected *DoHDAC* genes were induced by exogenous SA treatment (Fig. 10). All genes in roots were highly up-regulated after 2 h under SA treatment, while the expression of *DoHDA10* and *DoHDT4* in leaves was highly up-regulated after 24 h of SA treatment. Moreover, *cis*-acting elements in response to SA were found in these two genes (Table S2). ABA is a widely distributed plant hormone in land plants and plays an important role in plant development (Finkelstein, Gampala & Rock, 2002). After 4 h of ABA treatment, 11 *DoHDAC* genes, especially *DoHDA8*, *DoHDA10* and *DoHDT4*, were up-regulated in roots (Fig. 11), and *cis*-acting elements in response to ABA were found in the promoters of *DoHDA8* and *DoHDT4* (Table S2). After 24 h of treatment with MeJA, 12 *DoHDAC* genes in roots were up-regulated and two *DoHDAC* genes were down-regulated (Fig. 12). Interestingly, we found that after 2 h of MeJA treatment, *DoHDA10* and *DoHDT4* were significantly up-regulated in stems and leaves (> 9-fold), and *cis*-acting elements in response to MeJA were found in the promoters of these two genes (Table S2). These results indicate that *DoHDT4* might be involved in GA_3_-, SA-, ABA- and MeJA-mediated signal transduction pathways, *DoHDA10* might be involved in GA_3_-, SA- and MeJA-mediated signal transduction pathways, and *DoHDA8* might be involved in ABA-mediated signal transduction pathways.

### Expression analysis of *DoHDAC* genes in response to abiotic stresses

**Figure 13:**
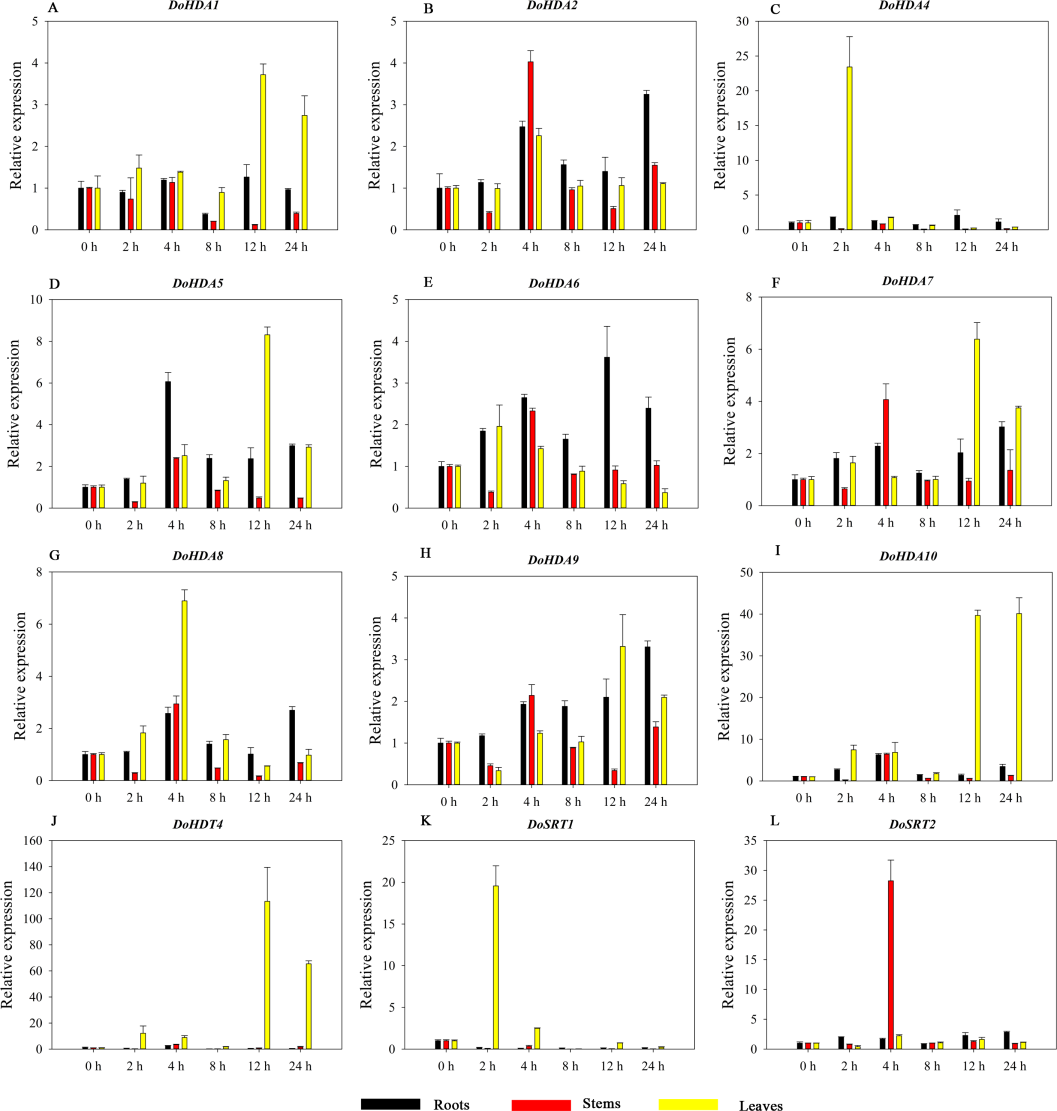
Quantitative real-time reverse transcriptase-PCR analysis of expression of selected *DoHDAC* genes at different hours of NaCl treatment in different tissues. A–L represent *DoHDA1*, *DoHDA2*, *DoHDA4*, *DoHDA5*, *DoHDA6*, *DoHDA7*, *DoHDA8*, *DoHDA9*, *DoHDA10*, *DoHDT4*, *DoSRT1*, *DoSRT2*, respectively. Error bars represent the standard error of the means of three independent replicates. Expression levels were calculated relative to 0 h of each organ.

**Figure 14:**
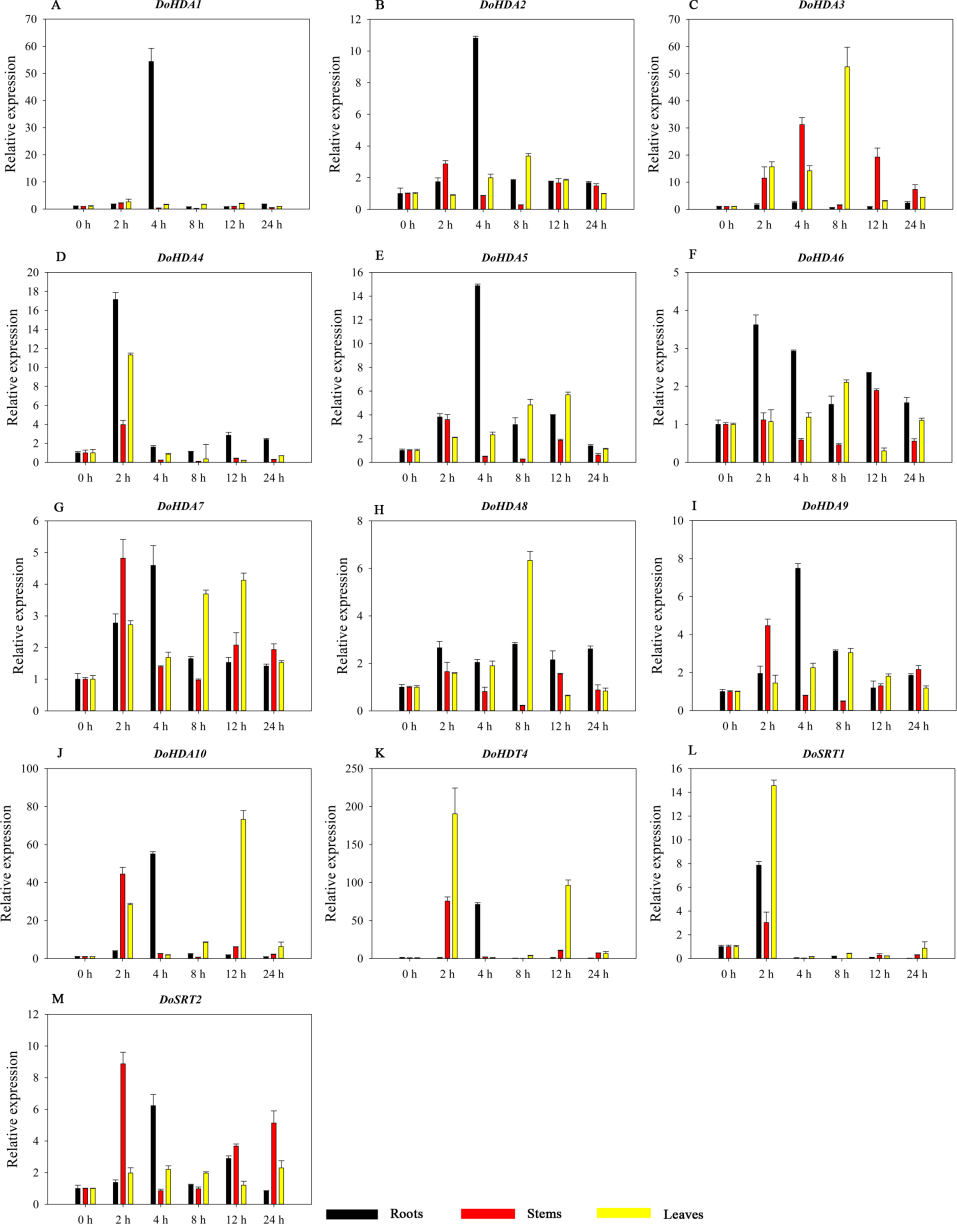
Quantitative real-time reverse transcriptase-PCR analysis of expression of selected *DoHDAC* genes at different hours of PEG-6000 treatment in different tissues. A–M represent *DoHDA1*, *DoHDA2*, *DoHDA3*, *DoHDA4*, *DoHDA5*, *DoHDA6*, *DoHDA7*, *DoHDA8*, *DoHDA9*, *DoHDA10*, *DoHDT4*, *DoSRT1*, *DoSRT2*, respectively. Error bars represent the standard error of the means of three independent replicates. Expression levels were calculated relative to 0 h of each organ.

**Figure 15:**
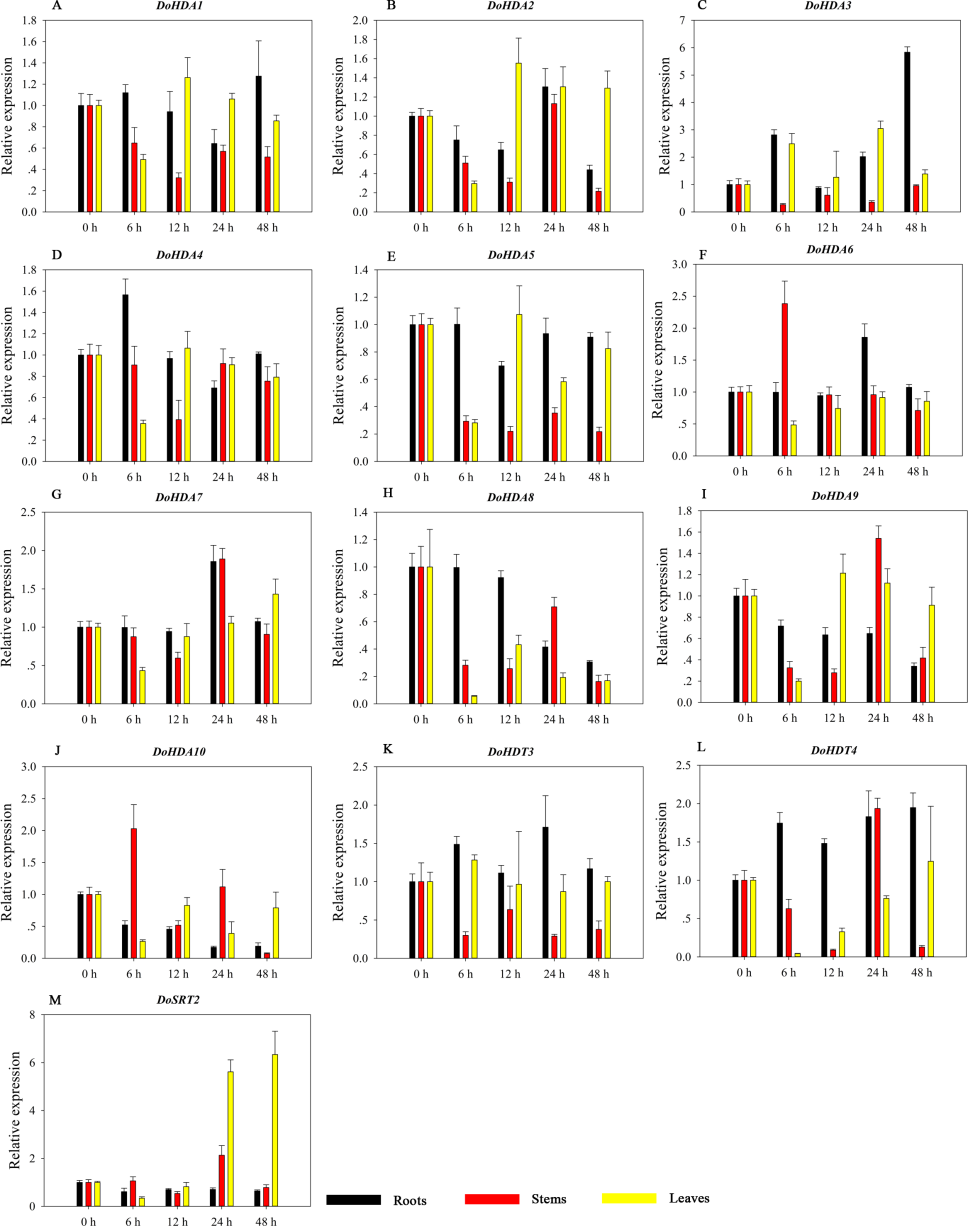
Quantitative real-time reverse transcriptase-PCR analysis of expression of selected *DoHDAC* genes at different hours of cold (4 °C) treatment in different tissues. A–M represent *DoHDA1*, *DoHDA2*, *DoHDA3*, *DoHDA4*, *DoHDA5*, *DoHDA6*, *DoHDA7*, *DoHDA8*, *DoHDA9*, *DoHDA10*, *DoHDT3*, *DoHDT4*, *DoSRT2*, respectively. Error bars represent the standard error of the means of three independent replicates. Expression levels were calculated relative to 0 h of each organ.

HDAC proteins appear to activate positive and negative responses in salinity stress, with some discrepancies. For example, AtHDA9 and AtHD2D negatively regulate the salt response, whereas AtHDA6 and AtHD2C positively regulate it (Ueda et al., 2017). After salt (NaCl) treatment, most of the selected *DoHDAC* genes were highly up-regulated in leaves, especially *DoHDA10* and *DoHDT4*, which showed similar expression patterns (Fig. 13). After PEG treatment, most genes were highly up-regulated in roots, stems and leaves, especially *DoHDA10* and *DoHDT4*, which showed similar expression patterns (Fig. 14). In addition, *cis*-acting elements involved in responsiveness to drought were found in the promoters of *DoHDA10* and *DoHDT4* (Table S2). The drought stress response in plants is related to the status of histone acetylation. In response to drought stress, the level of acetylation of histone H3K9 increased in drought-responsive genes (Kim et al., 2008). Drought stress significantly induced four *HAT* genes in rice (*OsHAC703*, *OsHAG703*, *OsHAF701*, and *OsHAM701*) (Fang et al., 2014). However, in this study, the up-regulated expression of *DoHDAC* genes under drought stress may be related to the inhibited expression of drought-insensitive genes. *AtHD2C*-overexpressing *A. thaliana* plants showed ABA insensitivity, reduced transpiration, and enhanced tolerance to drought stress (Sridha & Wu, 2006). After low-temperature treatment, the selected *DoHDAC* genes were weakly up-regulated in roots, stems and leaves (Fig. 15). This is consistent with the expression of most *HDAC* genes in rice being regulated by salt and drought, but less affected by cold (Hu et al., 2009). Therefore, *DoHDA10* and *DoHDT4* might play an important role in the regulation of salt and drought stress responses during the growth of *D. officinale* plants.

## Discussion

Plant HDAC proteins are a large family composed of multiple gene members. A variety of HDAC proteins have different subcellular localization and expression patterns, indicating that they have diverse functions. Reversible changes in histone deacetylation and acetylation play a crucial role in regulating gene expression involved in various developmental processes and plant responses to abiotic stress (Liu et al., 2014). Plant HDAC proteins have been identified and characterized in some plant species such as *A. thaliana*, rice, soybean, tomato and others. For instance, a total of 18 AtHDAC proteins have been identified, 12 of which belong to the RPD3/HDA1 family, four belong to the HD2 family, and two belong to the SIR2 family (Pandey et al., 2002; Wang et al., 2014). Furthermore, at least 18 HDAC proteins have been identified in the rice genome, 14 of which belong to the RPD3/HDA1 family, two belong to the HD2 family, and two belong to the SIR2 family (Hu et al., 2009). In this study, we identified 14 HDAC proteins in *D. officinale* by using bioinformatics analysis. However, the number of members of the HDAC proteins in *D. officinale* identified was less than that in *A. thaliana* and rice. The other DoHDAC proteins need to be identified and characterized in a future study.

Histone post-translational modifications (PTMs) are a major regulator of gene transcription. A number of PTMs are located within interfaces between H3/H4 tetramers and/or H2A/H2B dimers, such as the acetylation of H4(K91) (Zhang et al., 2003). Shortly after synthesis, histones H3 and H4 are acetylated by histone acetyltransferase 1/2 in the cytoplasm and then imported into the nucleus together with the histone acetyltransferase 1 complex. In the nucleus, H3-H4 dimers or tetramers are recognized by chaperones such as CAF-1, Asf1 and Hif1, perhaps through their acetylation markers and are deposited together with histone H2A-H2B dimers onto newly replicated DNA to form nucleosomes, and then rapidly deacetylated by histone deacetylase (Shahbazian & Grunstein, 2007). Moreover, some researches have shown that almost all acetyl-lysine-binding, bromodomain proteins (acetyl-lysine readers) are localized in the nucleus, and many of them are directly involved in the regulation of transcription (Narita, Weinert & Choudhary, 2019). Therefore, most HDAC proteins were localized in the nucleus in this study, which may be related to their function of removing acetyl groups from histones. The localization of HDAC proteins are also diverse in different environments. Some HDAC proteins are shuttled between the cytoplasm and the nucleus. For example, AtHDA15 shuttled from the cytoplasm to the nucleus in the presence of light, but exported out of the nucleus in darkness, implying that it could participate in the light signaling pathway (Alinsug et al., 2012). Moreover, the localization of HDAC proteins is variable in different plant tissues. For an instance, in anthers/endosperm, maize ZmHDA108 was localized in the nucleus and cytoplasm, while in shoot apexes, it was mainly found in the cytoplasm (Varotto et al., 2003). The green fluorescence of the DoHDA7-GFP and DoHDA9-GFP fusions were observed in the nucleus by *Agrobacterium*-mediated transient transformation used in onion epidermal cells (Fig. 6). Similarly, AtHDA6 and AtHDA19 was localized in the nucleus, which was consistent with the roles of these proteins as HDAC proteins (Kurita et al., 2019; Wu et al., 2008; Zhou et al., 2005). It should be noted, however, that the subcellular localization assays in the present study were performed using onion epidermal cells, and therefore, it is entirely possible that *D. officinale*-specific interactions could affect the localization pattern *in situ*.

Zinc finger proteins are a class of important transcription factors that regulate plant cell development (Sun et al., 2015). According to the number and position of cysteine residues and histidine residues, which bind zinc ions in the secondary structure of fingers, zinc finger proteins are classified into several types: C_2_H_2_, C_2_C_2_, C_2_HC, C_2_C_2_C_2_C_2_, and C_2_HCC_2_C_2_ (Ciftci-Yilmaz & Mittler, 2008). Among them, the C_2_H_2_-type zinc finger proteins are one of the most important groups and are involved in downstream gene regulation (Sakamoto et al., 2000; Tague, Gallant & Goodman, 1996). They are mainly involved in regulating biological processes such as plant branch and flower development, stress response and hormone signaling pathways (Sun et al., 2015). In this study, a typical C_2_H_2_-type zinc finger domain was found on the C-terminus of DoHDT3 (Fig. 2). Therefore, DoHDT3 might interact with other proteins to regulate plant development and stress response. AtHDA6 and AtHDA19 are the most well-studied RPD3/HDA1 family of HDAC proteins in plants, and they play a vital role in the growth and development of plants (Alinsug, Yu & Wu, 2009). Flowering is vital for plants to complete the life cycle and produce offspring. AtHDA6 and AtHDA19 are global repressors involved in flowering or flower development (Alinsug, Yu & Wu, 2009). Due to the high sequence similarity between DoHDA9 and AtHDA6 and between DoHDA7 and AtHDA19, DoHDA7 and DoHDA9 may have similar functions in regulating plant development as their homologs in *A. thaliana*. For example, when *AtHDA6* is suppressed, the expression of jasmonate-responsive genes is down-regulated (Wu et al., 2008). Similarly, after MeJA treatment, the expression level of *DoHDA9* was up-regulated in roots (Fig. 12). *AtHDA19* plays a pivotal role in tolerance to salinity stress (Ueda et al., 2017). Correspondingly, after NaCl treatment, the expression level of *DoHDA7* was up-regulated in stems and leaves (Fig. 13). The expression of *HDAC* genes occurs in response to stresses and is regulated by stress-related hormones like SA, JA or ABA (Fu, Wu & Duan, 2007; Hu et al., 2009). ABA is the plant hormone most directly involved in stress signal transduction (Xiong, Schumaker & Zhu, 2002). JA generally inhibits plant growth and promotes defense responses against insect pests and pathogens while ABA is involved in the water stress response, regulating plant water balance and osmotic stress tolerance (Ma et al., 2013). After ABA treatment, the expression levels of *AtHD2C*, which is a synonym of *AtHDT3*, *OsHDT701*, *OsHTD702*, *OsSRT701* and *OsSRT702* were down-regulated (Fu, Wu & Duan, 2007; Sridha & Wu, 2006). In contrast, the expression levels of *DoHDA10*, *DoHDT4*, and *DoSRT1* were significantly up-regulated in roots after ABA treatment and in leaves after NaCl treatment (Figs. 11 & 13), so they may affect the expression level of some stress-responsive genes. In addition to their functions in plant development and stress responses, HDAC proteins are also involved in cellular processes such as cell death. OsSRT1 in rice and NtHD2a and NtHD2b in tobacco act as negative regulators of cell death (Bourque et al., 2011; Huang et al., 2007). Programmed cell death (PCD) in the maize aleurone layer is induced by GA, and HDAC activity is required for this process. HDAC activity gradually increased relative to histone acetyltransferase (HAT) activity, leading to a global decrease in histone H3 and H4 acetylation levels during PCD after treatment with GA (Hou et al., 2017). These data further prove that the *DoHDAC* genes play an important role in the growth and development of *D. officinale* and in response to environmental stresses. Further research is required to identify proteins that interact with DoHDAC proteins and their global targets in *D. officinale*.

## Conclusions

We comprehensively identified and analyzed the *HDAC* genes from *D. officinale*. We performed a phylogenetic analysis, as well as analyses of conserved motifs, *cis*-acting elements, gene structure, protein interactions, and subcellular localization of some *DoHDAC* genes. These results help to elucidate the classification and function of the *DoHDAC* genes. Expression pattern analysis in different tissues showed that the *DoHDAC* genes were widely expressed in roots, stems, leaves and flower buds. The expression pattern of *DoHDAC* genes under abiotic stresses and various phytohormone treatments show that some *DoHDAC* genes were modulated by abiotic stresses such as salt, cold and drought, as well as plant hormones such as GA_3_, SA, ABA and MeJA. In summary, this information about the *HDAC* genes in *D. officinale* helps to reveal their role in epigenetic regulation of plant growth, development, and response to stresses.

##  Supplemental Information

10.7717/peerj.10482/supp-1 Supplemental Information 1 File 1: nucleotide and amino acid sequences. File 2: FKPM values of DoHDAC genes. File 3: qRT-PCR DATA summary

10.7717/peerj.10482/supp-2 Supplemental Information 2 Multiple sequence alignment of HDAC proteins in *D. officinale*, *O. sativa* and *A. thaliana* Asterisks represent active sites. Similar amino acids are shaded in gray. The same amino acids are shaded in black.

10.7717/peerj.10482/supp-3 Supplemental Information 3 Primers used for qRT-PCR.

10.7717/peerj.10482/supp-4 Supplemental Information 4 Predicted *cis.*-acting elements of *DoHDAC* promoters

10.7717/peerj.10482/supp-5 Supplemental Information 5 The mapping of homolog in *D. officinale.* and *A. thaliana.*

10.7717/peerj.10482/supp-6 Supplemental Information 6 Annotation of interactive proteins with DoHDAC proteins

## Additional Information and Declarations

### Data Deposition

The following information was supplied regarding data availability: The raw data are available in the Supplemental File.
