# Supplementary figures and images for: Identification of histone deacetylase genes in Dendrobium officinale and their expression profiles under phytohormone and abiotic stress treatments
Source: PeerJ. 2020 Dec 15;8:e10482. doi: 10.7717/peerj.10482 (PMC7747690; doi:10.7717/peerj.10482)

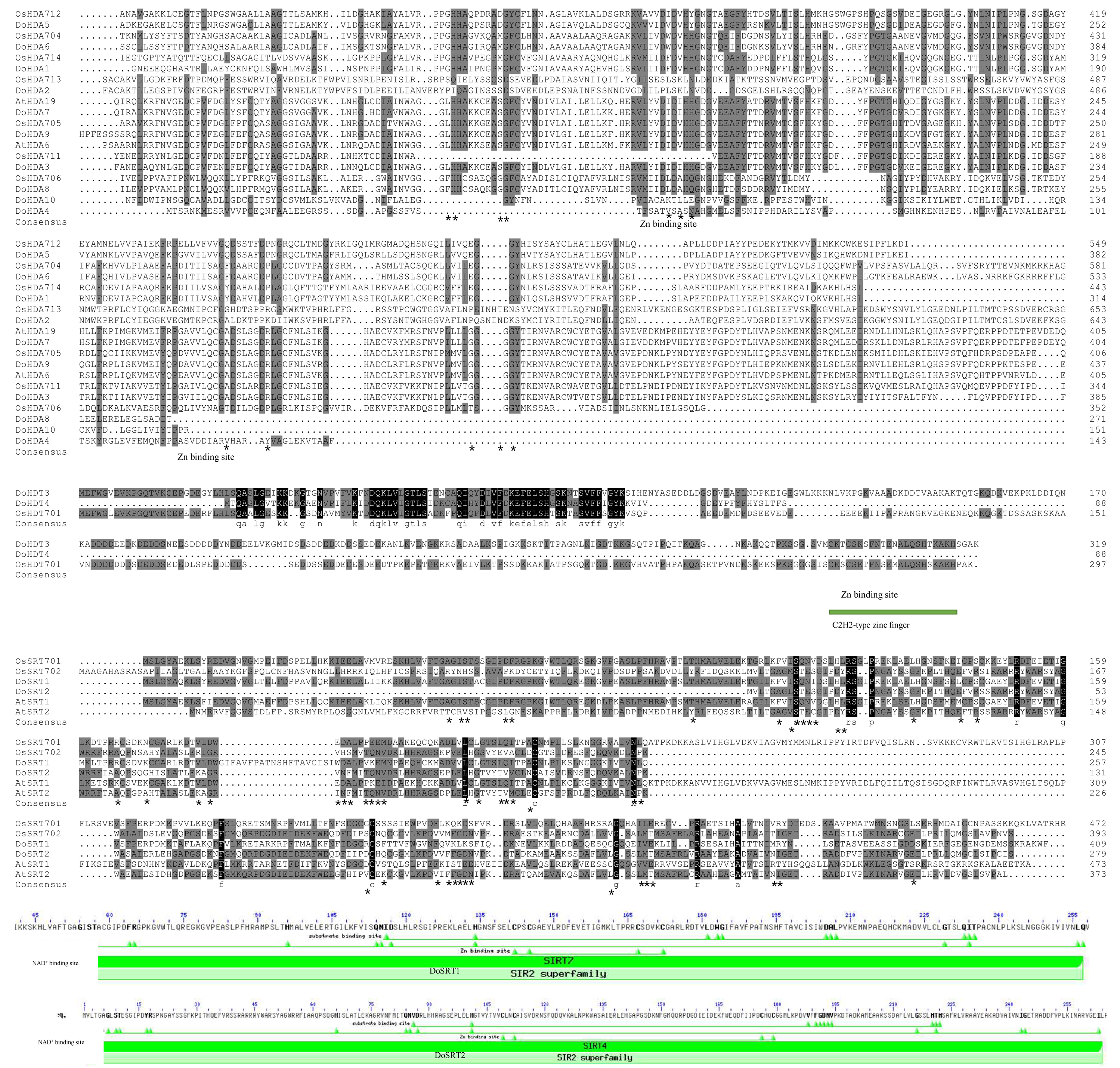

Supplement: Supplemental Information 2 — Asterisks represent active sites. Similar amino acids are shaded in gray. The same amino acids are shaded in black. [file peerj-08-10482-s002.png]
