# Supplementary material for: Identification of histone deacetylase genes in Dendrobium officinale and their expression profiles under phytohormone and abiotic stress treatments
Source: PeerJ. 2020 Dec 15;8:e10482. doi: 10.7717/peerj.10482 (PMC7747690; doi:10.7717/peerj.10482)
Supplement: Supplemental Information 3 [file peerj-08-10482-s003.docx]

| Gene | Forward primer sequence (5′→3′) | Reverse primer sequence (5′→3′) |
| --- | --- | --- |
| *Actin* | TCCCAAGGCAAACAGAGAAA | GGCCACTAGCATATAGGGAAAG |
| *DoHDA1* | GAGGGTCTGGTGATTATGCTATG | TAGCCAGCTGAGACGAGAATA |
| *DoHDA2* | CCTTTGGTGGATTCTCGTGATA | CCATCCTGCTCTCCCAAATAA |
| *DoHDA3* | TCATGGTGATGGAGTTGAAGAG | GTGCCCGGAAAGAACAAATC |
| *DoHDA4* | ACCACATGATGCACGTATCC | CAAACGCCTCAAGAGCATTAAC |
| *DoHDA5* | CCAACTATCCCGCCTTCTATC | CGTTGCATGGAGGCAATATG |
| *DoHDA6* | CAGACTGCTGGAGCCAATAA | GTCGGTGCAAAGAAACATACAG |
| *DoHDA7* | ATAGGCTCGGCTGCTTTAAC | CCGCCACCACCAAGTAATAA |
| *DoHDA8* | CAAGGGAATGGCCATGAAAC | CCTCCTTGCCTCGTAATCTAAA |
| *DoHDA9* | CAGGATAGACCACCAAAGACAG | ATAACTCTCGCCATCCCAAAG |
| *DoHD10* | TCATCGACTGGATCCCTAACT | GCTCTTTAGCATAACCGAACAATC |
| *DoHDT3* | CGAACCTGGAGATGAAGGATAC | TGTAGAGAGGGTCCCAAGAA |
| *DoHDT4* | GGCTTCGCTTGGTGTAACTA | GGTTCCAATGACTAGCTTCTGA |
| *DoSRT1* | TAGGGACACGGTTCTTGATTG | CTGGCAAAGCATCCCAAATC |
| *DoSRT2* | AGCCCAAATGGAGCGTATAG | ACCTTCTCCATCCAGCATAAC |
