# Supplementary material for: Identification of histone deacetylase genes in Dendrobium officinale and their expression profiles under phytohormone and abiotic stress treatments
Source: PeerJ. 2020 Dec 15;8:e10482. doi: 10.7717/peerj.10482 (PMC7747690; doi:10.7717/peerj.10482)
Supplement: Supplemental Information 4 [file peerj-08-10482-s004.docx]

| Site Name | Sequence (5′→3′) | Function | Gene (numbers of *cis*-acting elements) |
| --- | --- | --- | --- |
| 3-AF3 binding site | CACTATCTAAC | part of a conserved DNA module array (CMA3) | *DoSRT1* (1) |
| A-box | CCGTCC | *cis*-acting regulatory element | *DoHDA5* (1), *DoHDA8* (1), *DoHDA10* (2), *DoHDT3* (1), *DoSRT2* (1) |
| A-box | AATAACAAACTCC | sequence conserved in α-amylase promoters | *DoHDA7* (1) |
| AAAC-motif | CAATCAAAACCT | light responsive element | *DoHDA5* (1) |
| AACA_motif | TAACAAACTCCA | involved in endosperm-specific negative expression | *DoHDA7* (1) |
| ABRE | ACGTG | *cis*-acting element involved in abscisic acid responsiveness | *DoHDA1* (3), *DoHDA2* (2), *DoHDA4* (2), *DoHDA5* (2), *DoHDA8* (1), *DoHDA9* (1), *DoHDT4* (1) |
| ACA-motif | AATCACAACCATA | part of gapA in (gapA-CMA1) involved with light responsiveness | *DoSRT1* (1) |
| ACE | CTAACGTATT | *cis*-acting element involved in light responsiveness | *DoHDA5* (1) |
| AE-box | AGAAACAA | part of a module for light response | *DoHDA4* (1), *DoHDA10* (1), *DoSRT1* (1), *DoSRT2* (2) |
| ARE | AAACCA | *cis*-acting regulatory element essential for the anaerobic induction | *DoHDA1* (4), *DoHDA3* (2), *DoHDA4* (2), *DoHDA5* (2), *DoHDA6* (2), *DoHDA7* (2), *DoHDA9* (1), *DoHDA10* (1), *DoHDT3* (3), *DoHDT4* (5), *DoSRT1* (2), *DoSRT2* (1) |
| AT1-motif | AATTATTTTTTATT | part of a light responsive module | *DoHDA5* (1) |
| AT-rich sequence | TAAAATACT | element for maximal elicitor-mediated activation (2 copies) | *DoHDA3* (1) |
| AT-rich element | ATAGAAATCAA | binding site of AT-rich DNA binding protein (ATBP-1) | *DoHDA8* (1), *DoHDT3* (1) |
| ATC-motif | AGTAATCT | part of a conserved DNA module involved in light responsiveness | *DoHDA5* (1), *DoHDA9* (1) |
| ATCT-motif | AATCTAATCC | part of a conserved DNA module involved in light responsiveness | *DoHDA7* (1), *DoHDA9* (2) |
| AuxRR-core | GGTCCAT | *cis*-acting regulatory element involved in auxin responsiveness | *DoHDA7* (1), *DoHDA8* (1), *DoHDA9* (1), *DoHDT3* (1) |
| Box 4 | ATTAAT | part of a conserved DNA module involved in light responsiveness | *DoHDA1* (2), *DoHDA2* (2), *DoHDA3* (4), *DoHDA5* (2), *DoHDA6* (1), *DoHDA7* (3), *DoHDA8* (3), *DoHDA9* (2), *DoHDT3* (1), *DoHDT4* (3), *DoSRT1* (1), *DoSRT2* (2) |
| Box III | ATCATTTTCACT | protein binding site | *DoHDA3* (1) |
| CAT-box | GCCACT | *cis*-acting regulatory element related to meristem expression | *DoHDA1* (1), *DoHDA3* (1), *DoHDA4* (1), *DoHDA5* (1), *DoHDA9* (1), *DoHDA10* (2), *DoHDT3* (1), *DoHDT4* (1) |
| CCAAT-box | CAACGG | MYBHv1 binding site | *DoHDA7* (1), *DoSRT1* (1), *DoSRT2* (1) |
| CGTCA-motif | CGTCA | *cis*-acting regulatory element involved in MeJA-responsiveness | *DoHDA1* (3), *DoHDA3* (4), *DoHDA4* (1), *DoHDA5* (1), *DoHDA7* (1), *DoHDA10* (1), *DoHDT3* (3), *DoHDT4* (1), *DoSRT2* (6) |
| G-box | ACGTG | *cis*-acting regulatory element involved in light responsiveness | *DoHDA1* (3), *DoHDA2* (2), *DoHDA8* (2), *DoHDA9* (1), *DoHDA10* (1), *DoHDT3* (2) |
| GA-motif | ATAGATAA | part of a light-responsive element | *DoHDA7* (1), *DoHDA8* (1), *DoHDA9* (3), *DoSRT2* (1) |
| GC-motif | CCCCCG | enhancer-like element involved in anoxic-specific inducibility | *DoHDA2* (1) |
| Gap-box | CAAATGAA(A/G)A | part of a light-responsive element | *DoHDA2* (1), *DoHDA6* (1) |
| GARE-motif | TCTGTTG | gibberellin-responsive element | *DoHDA1* (2), *DoHDA5* (1), *DoHDA6* (1), *DoSRT2* (1) |
| GATA-motif | GATAGGG | part of a light-responsive element | *DoHDA1* (1), *DoHDA6* (1), *DoHDA8* (1), *DoHDA10* (2), *DoSRT2* (2) |
| GCN4_motif | TGAGTCA | *cis*-regulatory element involved in endosperm expression | *DoHDA5* (2), *DoHDA6* (1), *DoHDA10* (1) |
| GT1-motif | GGTTAA | light-responsive element | *DoHDA3* (1), *DoHDA5* (2), *DoHDA6* (3), *DoHDA7* (2), *DoHDA9* (1), *DoHDT3* (3), *DoSRT1* (3), *DoSRT2* (1) |
| I-box | AGATAAGG | part of a light-responsive element | *DoHDA4* (1), *DoHDA9* (2) |
| LAMP-element | CTTTATCA | part of a light-responsive element | *DoHDA5* (1) |
| LTR | CCGAAA | *cis*-acting element involved in low-temperature responsiveness | *DoHDA1* (1), *DoHDA3* (1), *DoHDA5* (1), *DoHDA6* (1), *DoHDT3* (1) |
| MBS | CAACTG | MYB binding site involved in drought-inducibility | *DoHDA3* (2), *DoHDA8* (1), *DoHDA9* (3), *DoHDA10* (1), *DoHDT3* (1), *DoHDT4* (1), *DoSRT2* (1) |
| NON-box | AGATCGACG | *cis*-acting regulatory element related to meristem-specific activation | *DoHDA10* (1) |
| MRE | AACCTAA | MYB binding site involved in light responsiveness | *DoHDA1* (1), *DoHDA4* (2), *DoHDA5* (2), *DoHDA6* (1), *DoHDA8* (1), *DoHDT3* (2), *DoSRT1* (3), *DoSRT2* (1) |
| O2-site | GTTGACGTGA | *cis*-acting regulatory element involved in zein metabolism regulation | *DoHDA6* (1), *DoHDA9* (2), *DoHDT3* (1), *DoSRT1* (1), *DoSRT2* (1) |
| P-box | CCTTTTG | gibberellin-responsive element | *DoHDA4* (1), *DoHDA7* (1), *DoHDA8* (1), *DoHDT4* (1) |
| Sp1 | GGGCGG | light-responsive element | *DoHDA4* (1), *DoHDA10* (3), *DoHDT3* (1) |
| TATC-box | TATCCCA | *cis*-acting element involved in gibberellin responsiveness | *DoHDA8* (1), *DoHDA9* (1), *DoHDA10* (1), *DoHDT3* (1) |
| TC-rich repeats | GTTTTCTTAC | *cis*-acting element involved in defense and stress responsiveness | *DoHDA2* (1), *DoHDA5* (1), *DoHDA6* (1), *DoHDA7* (1), *DoHDT4* (1), *DoSRT1* (1), *DoSRT2* (1) |
| TCCC-motif | TCTCCCT | part of a light-responsive element | *DoHDA3* (1), *DoHDA9* (1), *DoHDT4* (2) |
| TCA-element | CCATCTTTTT | *cis*-acting element involved in salicylic acid responsiveness | *DoHDA6* (2), *DoHDA8* (2), *DoHDA9* (1), *DoHDA10* (1), *DoHDT4* (3) |
| TCT-motif | TCTTAC | part of a light-responsive element | *DoHDA1* (3), *DoHDA2* (2), *DoHDA3* (3), *DoHDA4* (2), *DoHDA6* (4), *DoHDA7* (2), *DoHDA8* (1), *DoSRT1* (3), *DoSRT2* (2) |
| TGA-element | AACGAC | auxin-responsive element | *DoHDA9* (1), *DoHDA10* (2), *DoHDT3* (2), *DoSRT1* (2) |
| TGACG-motif | TGACG | *cis*-acting regulatory element involved in MeJA-responsiveness | *DoHDA1* (3), *DoHDA3* (4), *DoHDA4* (1), *DoHDA5* (1), *DoHDA7* (1), *DoHDA10* (1), *DoHDT3* (3), *DoHDT4* (1), *DoSRT1* (3), *DoSRT2* (6) |
| WUN-motif | AAATTTCCT | wound-responsive element | *DoHDA6* (1) |
| circadian | CAAAGATATC | *cis*-acting regulatory element involved in circadian control | *DoHDA5* (1) |
| chs-CMA2a | TCACTTGA | part of a light-responsive element | *DoHDA10* (1), *DoHDT3* (1) |
